# Association of high-normal blood pressure defined by the 2023 European Society of Hypertension guideline with mortality in the Chinese population: a nationwide, population-based, prospective study of 3.6 million adults

**DOI:** 10.1186/s12916-025-04055-5

**Published:** 2025-04-16

**Authors:** Zhiwei Li, Mengmeng Liu, Bowang Chen, Yuelin Wu, Hui Jia, Ruirui Geng, Yixiao Wang, Xiaoyan Zhang, Yang Yang, Jianlan Cui, Jiapeng Lu, Zhiping Guo, Xi Li, Weili Zhang

**Affiliations:** 1 National Clinical Research Center of Cardiovascular Diseases, National Center for Cardiovascular Diseases, Fuwai Hospital, Chinese Academy of Medical Sciences and Peking Union Medical College, 167 Beilishi Road, Beijing, 100037 People’s Republic of China; 2 Central China Subcenter of National Center for Cardiovascular Diseases, Henan Cardiovascular Disease Center, Fuwai Central-China Cardiovascular Hospital, Central China Fuwai Hospital of Zhengzhou University, Zhengzhou, 450000 People’s Republic of China; 3 Henan Key Laboratory of Chronic Disease, Fuwai Central China Cardiovascular Hospital, Zhengzhou, 450000 People’s Republic of China; 4 Shenzhen Clinical Research Center for Cardiovascular Diseases, Fuwai Hospital Chinese Academy of Medical Sciences, Shenzhen, Shenzhen, 518000 People’s Republic of China

**Keywords:** High-normal blood pressure, Trajectory pattern, All-cause mortality, Cardiovascular mortality

## Abstract

**Background:**

The relationship between high-normal blood pressure (BP) and mortality lacks high-quality evidence based on large population cohorts. This study aims to comprehensively investigate the association of high-normal BP and its trajectory with all-cause and cause-specific mortality.

**Methods:**

In this community-based population cohort from the China Health Evaluation And risk Reduction Through nationwide teamwork (ChinaHEART) project, 3,598,940 participants aged 35–75 years with data for baseline BP were included. High-normal BP was defined as a systolic BP (SBP) of 130–139 mmHg and/or a diastolic BP (DBP) of 85–89 mmHg at baseline. Overall, 78,130 participants with three or more BP measurements were included in the trajectory pattern analysis during the follow-up. Four BP change trajectory patterns were identified.

**Results:**

For the baseline BP analysis, compared with the optimal BP group (SBP < 120 mmHg and DBP < 80 mmHg [18.1%]), participants with high-normal BP (18.7%) had an increase of 4% in all-cause mortality risk (hazard ratio [HR] 1.04, 95% confidence interval [CI] 1.01–1.07) and an increase of 28% in cardiovascular disease (CVD) mortality risk (HR 1.28, 95% CI 1.21–1.34), with the greatest increase in mortality risk observed for hemorrhagic stroke (HR 1.75, 95% CI 1.55–1.98). Among the BP trajectory patterns, compared with participants with optimal-stable BP, those with high-normal-increasing BP had an increase of 35% in all-cause mortality risk (HR 1.35, 95% CI 1.07–1.70) and an increase in CVD mortality risk of 57% (HR 1.57, 95% CI 1.11–2.24), with the greatest increase in mortality risk also observed for hemorrhagic stroke (HR 3.75, 95% CI 1.50–9.34). Approximately 0.7% and 1.6% of all-cause mortality was attributable to high-normal BP at baseline and the high-normal-increasing BP trajectory pattern, respectively.

**Conclusions:**

Individuals with high-normal BP at baseline exhibited a significantly elevated mortality risk and especially for risk of hemorrhagic stroke mortality during the follow-up. This positive association may be mainly attributed to the “high-normal-increasing” BP change over time.

**Supplementary Information:**

The online version contains supplementary material available at 10.1186/s12916-025-04055-5.

## Background

Currently, universally accepted criteria for defining high-normal blood pressure (BP), also known as prehypertension or elevated BP, are lacking on a global scale. Various researchers have proposed different definitions for high-normal BP. Some have defined high-normal BP as systolic BP (SBP) and diastolic BP (DBP) values falling within the ranges of 120–139 mmHg and 80–89 mmHg, respectively [1], while others have proposed SBP and DBP ranges of 130–139 mmHg and 80–89 mmHg, respectively [2, 3]. Owing to these varying definitions and differences in participant eligibility, prior study results regarding the association of high-normal BP with mortality and morbidity have been inconsistent [4–7]. A meta-analysis incorporating 13 cohort studies with a total of 870,678 participants indicated that the BP range of 120–139/80–89 mmHg was not associated with mortality; however, when the BP range was restricted to 130–139/85–89 mmHg, a significant association with mortality was observed [8]. In 2023, the European Society of Hypertension (ESH) defined high-normal BP as 130–139/85–89 mmHg [9].

A single BP measurement is insufficient to determine BP-related mortality risk. In a previous study, nearly half of individuals with a BP of 130–139/85–89 mmHg progressed to hypertension within 4 years [10]. However, most cohort studies have focused only on the association between baseline BP and mortality or morbidity at baseline [11, 12]. Trajectory assessment is needed to better reflect long-term BP patterns [13, 14], which is essential for better BP management.

On the basis of a national population cohort of 3.6 million Chinese adults, this study aims to comprehensively explore the association of high-normal BP (130–139/85–89 mmHg) and its trajectory pattern with all-cause and cause-specific mortality, thereby providing solid evidence and new insights into population-level BP management strategies.

## Methods

### Study population

The China Health Evaluation And risk Reduction Through nationwide teamwork (ChinaHEART) project is a government-supported public health program aimed at identifying and addressing cardiovascular disease (CVD) risk factors within community-based populations throughout China. The project’s methodology for sampling, participant recruitment, and frequency of follow-up visits is outlined in the supplementary materials (Additional file 1: Figure S1, Table S1). Other details of the project have been described previously [15]. In summary, between September 2014 and December 2022, a total of 359 study sites were selected, encompassing 141 urban districts and 212 rural counties across all 31 provinces of mainland China to ensure a diverse representation in terms of geographical location, demographics, economic status, and population composition. Eligible individuals aged 35–75 years who had resided in the community for at least 6 months were recruited for the study. The study was based on a dynamic cohort, with participants enrolled annually. To date, 12,323,531 participants have been invited to participate, with around 4.7 million ultimately enrolled in the ChinaHEART project. The Ethics Committee of Fuwai Hospital Chinese Academy of Medical Sciences approved the project (2014–574). All registered participants provided written informed consent. After enrollment, participants underwent a baseline assessment. If participants met the criteria for high CVD risk, they were invited to annual follow-up visits.

**Fig. 1:**
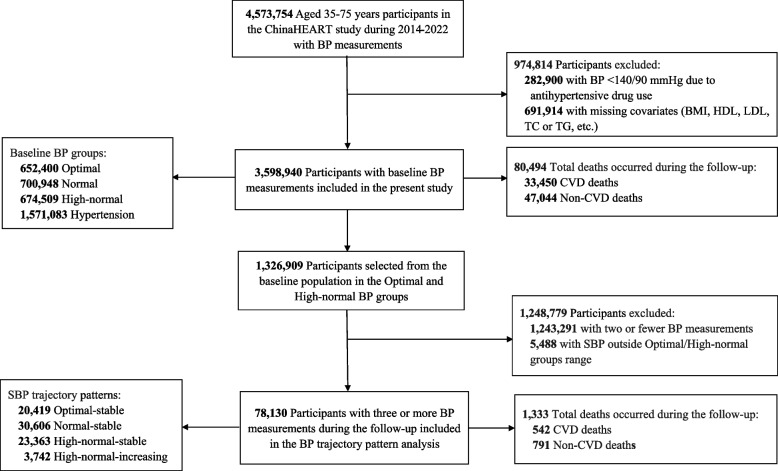
Flow chart of enrollment in the study population. Abbreviations: BP, blood pressure; BMI, body mass index; HDL, high-density lipoprotein; LDL, low-density lipoprotein; TC, total cholesterol; TG, triglyceride; CVD, cardiovascular disease. BP grouping criteria: 1) optimal: SBP < 120 mmHg and DBP < 80 mmHg; 2) normal: SBP:120–129 mmHg and DBP: 80–84 mmHg; 3) high-normal: SBP:130–139 mmHg and/or DBP: 85–89 mmHg; 4) hypertension: SBP ≥ 140 mmHg and/or DBP ≥ 90 mmHg

The flowchart of the present study is shown in Fig. 1. Briefly, participants from the ChinaHEART project spanning 2014–2022 with baseline SBP/DBP values above 90/60 mmHg were included. Participants with incomplete data for covariates, such as body mass index (BMI), total cholesterol, high-density lipoprotein (HDL) cholesterol, low-density lipoprotein (LDL) cholesterol, and triglyceride, for the analysis of the relationship between baseline BP and mortality were excluded. Additionally, participants with three or more BP measurements during follow-up were included in the trajectory pattern analysis.

### Data collection and variable definitions

During the initial evaluation, proficient interviewers conducted face-to-face interviews with every participant to collect information regarding socioeconomic characteristics (including educational background, annual household income, health insurance, marital status, and occupation), lifestyle (smoking and alcohol usage), and self-reported medical history using electronic questionnaires with a real-time logical check function.

The participants underwent blood tests to measure lipids and glucose in whole-blood samples using standardized devices. Total cholesterol, triglycerides, and HDL cholesterol were measured using a rapid lipid analyzer. LDL cholesterol was calculated using the Friedewald equation. Glucose was measured using a rapid blood glucose analyzer. The participants were in a fasting state if they had consumed their last meal at least 8 h prior to their appointment.

### BP measurement and classification

BP was measured twice at the right upper arm after a 5-min period of rest in the seated position. An electronic BP monitor was used for the measurements. If there was a difference of > 10 mmHg between the two SBP readings, a third measurement was obtained, and the average of the last two readings was calculated for the analysis.

The 2023 ESH guidelines for the management of arterial hypertension were used to categorize BP into four categories [9]: 1) optimal: SBP < 120 mmHg and DBP < 80 mmHg; 2) normal: SBP 120–129 mmHg and DBP 80–84 mmHg; 3) high-normal: SBP 130–139 mmHg and/or DBP 85–89 mmHg; 4) hypertension: SBP ≥ 140 mmHg and/or DBP ≥ 90 mmHg. To investigate the relationship between untreated BP and mortality, individuals with SBP < 140 mmHg and/or DBP < 90 mmHg with antihypertensive medication were excluded from the study.

**Fig. 2:**
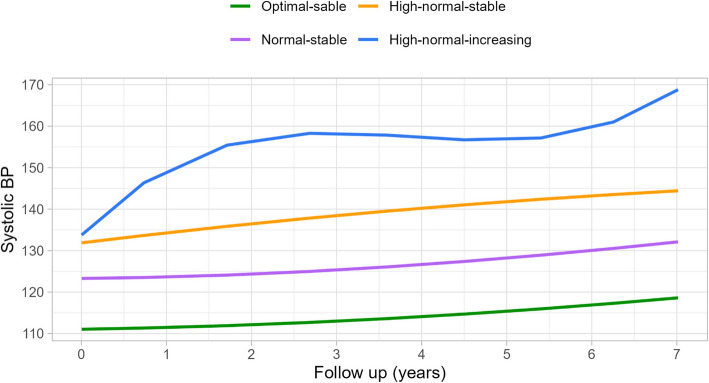
SBP trajectory patterns during the follow-up. Abbreviations: SBP, systolic blood pressure; The participants included in this study had at least three SBP measurements. Optimal-stable: *n* = 20,419 (26.5%); normal-stable: *n* = 30,606 (38.3%); high-normal-stable: *n* = 23,363 (29.6%); high-normal-increasing: *n* = 3,742 (5.6%). Follow-up years represented the time since baseline enrollment, where 0 represented the baseline measurement

To explore the association between the BP trajectory pattern during follow-up and mortality, four distinct BP trajectory patterns were identified on the basis of three or more BP measurements for each individual (Additional file 1: Table S2). We defined the naming of the trajectory groups based on the SBP trend during the follow-up visits. If the initial SBP was < 120 mmHg, it was defined as “optimal.” If the initial SBP fell between 120 and 130 mmHg, it was defined as “normal.” If the initial SBP was between 130 and 139 mmHg, it was defined as “high-normal.” During the follow-up visits, individuals whose SBP changes over time did not exceed the 20 mmHg threshold were classified as having a “stable trend,” whereas those with changes exceeding this threshold were classified as having an “increasing trend”. Similar to the definitions in the studies by Allen and Liu [14, 16]. Therefore, we defined the four trajectory pattern groups as follows: optimal-stable, normal-stable, high-normal-stable, and high-normal-increasing (Fig. 2).

### Study outcomes

All-cause mortality, CVD mortality, and non-CVD mortality were the study outcomes. The death records of all participants were collected through passive follow-up, where data links were established between the ChinaHEART cohort and the National Mortality Surveillance System and the Vital Registration of the Chinese Center for Disease Control and Prevention, covering urban and rural areas in 31 provinces of mainland China. Death records in the system were reported by health care institutions in real-time and subsequently checked against local residence records and health insurance records annually.

In the National Mortality Surveillance System and the Vital Registration of the Chinese Center for Disease Control and Prevention, the main causes of death are coded using the 10 th edition of the International Classification of Diseases (ICD- 10). The CVD mortality subtypes were hemorrhagic stroke (ICD- 10: I60–I62), ischemic stroke (ICD- 10: I63), and ischemic heart disease (IHD; ICD- 10: I20–I25). The non-CVD mortality subtypes were chronic obstructive pulmonary disease (COPD; ICD- 10: J41–J44) and neoplasms (ICD- 10: C00–D48).

### Statistical analysis

The characteristics of the participants were described according to the baseline BP groups or BP trajectory patterns during follow-up. Categorical variables were presented as percentages, while continuous variables were expressed as the mean (standard deviations [SD]) or median (interquartile range[IQR]). The incidence rate (per 1,000 person-years) of all-cause mortality, CVD mortality, and non-CVD mortality for participants in each baseline BP group and BP trajectory pattern group during follow-up was calculated.

In contrast to DBP, SBP is associated with mortality and morbidity in a variety of diseases [17–19]. Therefore, SBP was used to fit the trajectory pattern. Latent mixture modeling was used to identify the trajectories with similar underlying SBP patterns [20]. The data were analyzed using models that included linear, quadratic, and cubic terms for time. On the basis of the requirement for the number of participants in each trajectory group to be > 5% and the criterion of minimizing the Bayesian Information Criterion (BIC) value [16, 21, 22], four groups with cubic terms were used for the trajectory pattern analysis during follow-up.

The Cox proportional hazards model (for all-cause mortality) or the competing risk model (for CVD mortality and non-CVD mortality) was used to assess hazard ratios (HRs) between the baseline BP groups or BP trajectory patterns and mortality, with corresponding 95% confidence intervals (CIs). The models were adjusted for sex, age, region, level of education, annual household income, urbanity, smoking, alcohol consumption, BMI, total cholesterol, HDL, LDL, triglyceride, fasting blood glucose, medical history, dyslipidemia, anti-hypertensive drug use, and high CVD risk. The reference group for the baseline BP group analysis was defined as “optimal,” whereas “optimal-stable” was chosen as the reference group for the BP trajectory pattern analysis during follow-up. For the baseline BP analysis, the participants were followed from the date of enrollment until death or December 31, 2022, whichever came first. For the BP trajectory pattern analysis, the participants were followed from the date of the last BP measurement during follow-up to either the occurrence of death or December 31, 2022, whichever came first. In the trajectory analysis, the earliest enrolled participant was enrolled in 2014, while the latest enrolled participant was enrolled in 2020. The last date of BP measurement during follow-up was December 1, 2022.

To understand the attributable risk of mortality in patients with high-normal BP, the population attributable risk (PAR) for all-cause mortality, CVD mortality, and non-CVD mortality attributable to “high-normal BP” in the baseline BP group and “high-normal-increasing” in the BP trajectory pattern group was calculated using the Levin approach [23]. The equation is as follows:

$$PAR(\%)= \frac{\left(\frac{Exposure}{N}\right)*(HR-1)}{(\frac{Exposure}{N})*\left(HR-1\right)+1}$$

where *Exposure* represents the number of participants in the “high-normal” BP classification or the “high-normal-increasing” BP trajectory pattern group, *N* denotes the number of all participants in the study, and *HR* is the estimated HR based on the Cox proportional-hazards model or the competing risk model*.*

Pre-specified subgroup analyses by age and sex were conducted to identify susceptible populations. Several sensitivity analyses were also performed. First, the HRs were recalculated by excluding mortality events within the first 3 years of follow-up. Second, among the participants in ChinaHEART, those with identification numbers ending in 1, 3, 5, or 7 were chosen to form a sub-cohort for which more detailed information on physical activity and food intake was collected. These participants were selected for model refitting with additional adjustment for physical activity and dietary habits in the model. The absolute change in HR resulting from the sensitivity analysis relative to the primary analysis was calculated.

The *PROC TRAJ* macroprogram in SAS 9.4 (SAS Institute Inc., Cary, NC, US) and the *survival* and *tidycmprsk* packages in R 4.3.1 (R Foundation for Statistical Computing, Vienna, Austria) were used for the statistical analyses. Two-sided *P* values of < 0.05 were considered statistically significant.

## Results

### Participant characteristics

Overall, 3,598,940 participants were included in the baseline BP analysis, with a mean age of 56.0 ± 10.0 years. Of these participants, 40.1% (1,442,720) were male and 60.8% (2,189,361) lived in rural areas. Less than half (45.6%) had attained only primary school education or below, and 18.6% reported an annual household income of more than 50,000 RMB. Overall, 19.5% of the participants were current smokers, and 24.2% were current drinkers. The majority (55.3%) had a BMI of between 18 and 24 kg/m^2^ (Table 1).

**Table 1:** Characteristics of participants in ChinaHEART study with different BP group using 2023 ESH guideline

Characteristics	Overall (*N* = 3,598,940)	Optimal (*N* = 652,400)	Normal (*N* = 700,948)	High-normal (*N* = 674,509)	Hypertension (*N* = 1,571,083)
SBP/DBP, mmHg		< 120 and < 80	120–129 and 80–84	130–139 and/or 85–89	> = 140 and/or > = 90
SBP, mm Hg, mean (SD)	136.55 (20.2)	111.19 (6.2)	123.56 (4.2)	132.83 (4.8)	148.83 (10.8)
DBP, mm Hg, mean (SD)	81.92 (10.8)	70.79 (4.9)	76.63 (5.4)	80.63 (6.2)	90.82 (5.6)
Men, n (%)	1,442,720 (40.1)	214,622 (32.9)	284,940 (40.7)	288,039 (42.7)	655,119 (41.7)
Age, year, mean (SD)	56.00 (10.0)	51.16 (9.7)	53.59 (9.9)	55.80 (9.8)	57.38 (8.7)
Age, year, n (%)					
35–44	531,287 (14.8)	186,093 (28.5)	144,495 (20.6)	95,230 (14.1)	105,469 (6.7)
45–54	1,099,905 (30.6)	243,356 (37.3)	246,450 (35.2)	217,567 (32.3)	392,532 (25.0)
55–64	1,107,540 (30.8)	147,852 (22.7)	190,746 (27.2)	210,786 (31.3)	558,156 (35.5)
65–75	860,208 (23.9)	75,099 (11.5)	119,257 (17.0)	150,926 (22.4)	514,926 (32.8)
Region, n (%)					
East China	1,122,218 (31.2)	184,790 (28.3)	209,409 (29.9)	213,021 (31.6)	514,998 (32.8)
Central China	840,327 (23.3)	139,422 (21.4)	154,991 (22.1)	157,425 (23.3)	388,489 (24.7)
West China	1,307,488 (36.3)	277,967 (42.6)	262,227 (37.4)	234,400 (34.8)	532,894 (33.9)
Northeast China	328,907 (9.1)	50,221 (7.7)	74,321 (10.6)	69,663 (10.3)	134,702 (8.6)
Education, n (%)					
Primary school and below	1,641,299 (45.6)	239,953 (36.8)	286,765 (40.9)	307,999 (45.7)	806,582 (51.3)
Middle school	1,148,403 (31.9)	216,840 (33.2)	234,510 (33.5)	222,205 (32.9)	474,848 (30.2)
High school and above	771,773 (21.4)	189,075 (29.0)	171,650 (24.5)	136,527 (20.2)	274,521 (17.5)
Unknown	37,465 (1.0)	6,532 (1.0)	8,023 (1.1)	7,778 (1.2)	15,132 (1.0)
Annual household income, RMB, n (%)					
< 10 k	614,424 (17.1)	90,509 (13.9)	105,873 (15.1)	113,782 (16.9)	304,260 (19.4)
10 k- 50 k	1982,603 (55.1)	350,826 (53.8)	387,511 (55.3)	376,771 (55.9)	867,495 (55.2)
> = 50 k	670,264 (18.6)	148,813 (22.8)	143,041 (20.4)	121,379 (18.0)	257,031 (16.4)
Unknown	331,649 (9.2)	62,252 (9.5)	64,523 (9.2)	62,577 (9.3)	142,297 (9.1)
Live in Rural, n (%)	2,189,361 (60.8)	374,102 (57.3)	411,081 (58.6)	409,753 (60.7)	994,425 (63.3)
Current smoker, n (%)	700,898 (19.5)	116,578 (17.9)	133,954 (19.1)	136,179 (20.2)	314,187 (20.0)
Current drinker, n (%)	871,938 (24.2)	139,203 (21.3)	158,797 (22.7)	164,773 (24.4)	409,165 (26.0)
BMI, kg/m^2^, mean (SD)	24.74 (3.4)	23.41 (3.0)	24.14 (3.1)	24.66 (3.2)	25.64 (3.5)
BMI, kg/m^2^, n (%)					
< 18	39,086 (1.1)	13,163 (2.0)	8,348 (1.2)	6,581 (1.0)	10,994 (0.8)
18–24	1,988,249 (55.3)	462,086 (70.9)	442,723 (63.2)	379,541 (56.3)	703,899 (44.8)
24–28	988,731 (27.5)	130,554 (20.0)	174,255 (24.9)	189,638 (28.2)	494,284 (31.5)
> = 28	578,626 (16.1)	45,896 (7.0)	74,637 (10.7)	97,872 (14.5)	360,221 (22.9)
Total cholesterol, mmol/L, mean (SD)	4.58 (1.1)	4.41 (1.00)	4.48 (1.01)	4.57 (1.0)	4.64 (1.08)
Total cholesterol, mmol/L, n (%)					
< 5.2	2,710,392 (75.3)	527,080 (80.8)	553,891 (79.0)	512,402 (76.0)	1,117,019 (71.1)
5.2–6.19	640,420 (17.8)	93,632 (14.4)	109,055 (15.6)	118,901 (17.6)	318,832 (20.3)
> = 6.2	248,128 (6.9)	31,688 (4.9)	38,002 (5.4)	43,206 (6.4)	135,232 (8.6)
HDL-cholesterol, mmol/L, mean (SD)	1.42 (0.4)	1.44 (0.4)	1.43 (0.4)	1.43 (0.4)	1.39 (0.4)
LDL-cholesterol, mmol/L, mean (SD)	2.44 (0.9)	2.34 (0.9)	2.39 (0.9)	2.45 (0.9)	2.48 (0.9)
Triglycerides, mmol/L, median (IQR)	1.58 (0.8)	1.42 (0.7)	1.50 (0.8)	1.56 (0.8)	1.7 (0.9)
Fasting blood glucose, mmol/L, mean (SD)	6.14 (1.7)	5.82 (1.4)	5.90 (1.5)	6.07 (1.6)	6.35 (1.8)
Fasting blood glucose, mmol/L, n (%)					
< 5.6	1,388,048 (38.6)	312,806 (47.9)	316,862 (45.2)	267,785 (39.7)	490,595 (31.2)
5.6–6.9	1,620,079 (45.0)	275,923 (42.3)	303,037 (43.2)	308,761 (45.8)	732,358 (46.6)
> = 7.0	590,813 (16.4)	63,671 (9.8)	81,049 (11.6)	97,963 (14.5)	348,130 (22.2)
Medical history, n (%)					
Cardiovascular disease	107,033 (3.0)	8,903 (1.4)	10,671 (1.5)	12,645 (1.9)	74,814 (4.8)
Stroke	73,094 (2.0)	4,976 (0.8)	6,435 (0.9)	7,926 (1.2)	53,757 (3.4)
Myocardial infarction	21,015 (0.6)	2,511 (0.4)	2,671 (0.4)	2,867 (0.4)	12,966 (0.8)
Hypertension	681,103 (18.9)	0 (0.0)	0 (0.0)	0 (0.0)	681,103 (43.4)
Diabetes mellitus	234,467 (6.5)	19,988 (3.1)	27,080 (3.9)	33,543 (5.0)	153,856 (9.8)
Dyslipidemia, n (%)	352,489 (9.8)	41,662 (6.4)	49,993 (7.1)	56,390 (8.4)	204,444 (13.0)
Anti-hypertensive drug use, n(%)	468,944 (13.0)	0 (0.0)	0 (0.0)	0 (0.0)	468,944 (29.8)
High CVD risk ^a^, n (%)	819,620 (22.8)	39,681 (6.1)	45,762 (6.5)	50,530 (7.5)	683,647 (43.5)
Mortality, n (%)					
Total mortality	80,494 (2.2)	8,611 (1.3)	11,265 (1.6)	13,092 (1.9)	47,526 (3.0)
CVD mortality	33,450 (0.9)	2,492 (0.4)	3,725 (0.5)	4,741 (0.7)	22,492 (1.4)
Non-CVD mortality	47,044 (1.3)	6,119 (0.9)	7,540 (1.1)	8,351 (1.2)	25,034 (1.6)

*Abbreviations*: *BP* Blood pressure, *ESH* European society of hypertension, *SD* Standard deviation, *SBP* Systolic blood pressure, *DBP* Diastolic blood pressure, *BMI* Body mass index (calculated as weight in kilograms divided by height in meters squared), *HDL* High-density lipoprotein, *LDL* Low-density lipoprotein, *IQR* interquartile range, *CVD* Cardiovascular disease

SI conversion factors: To convert HDL, LDL and total cholesterol to mg/dL, divide by 0.0259; Fasting blood glucose to mg/dL, divide by 0.0555; Triglycerides to mg/dL, divide by 0.0113

^a^High CVD risk should meet at least one of four criteria: (i) major cardiovascular events history (myocardial infarction, percutaneous coronary intervention, coronary artery bypass graft or stroke); (ii) a predicted CVD risk ≥ 20% based on World Health Organization(WHO)/International Society of Hypertension cardiovascular risk prediction charts [15]; (iii) severely abnormal blood lipid levels (LDL ≥ 4.14 mmol/L or HDL < 0.78 mmol/L); or (iv) severely high blood pressure (SBP > 160 mmHg or DBP > 100 mmHg)

In the baseline BP analysis, 674,509 participants had high-normal BP (SBP 130–139 mmHg and/or DBP 85–89 mmHg), accounting for 18.1% of the overall cohort. Compared with participants in the optimal BP group, those in the high-normal BP group were more likely to be men, older, living in rural areas, have a lower level of education or income, and have an unhealthy lifestyle and metabolic risk factors (*P* < 0.001 for all).

In the trajectory pattern analysis during follow-up, 78,130 participants with three or more BP measurements were included. Among them, 23,207 participants had three BP measurements, and 585 participants had up to nine measurements (Additional file 1: Table S2). Compared with participants in the optimal-stable group (Additional file 1: Table S3), those in the high-normal-increasing BP trajectory group were more likely to be men, older, living in rural areas, have a lower level of education or income, and have an unhealthy lifestyle and metabolic risk factors (*P* < 0.001 for all). During the modeling process, the average SBP value for each trajectory group was re-estimated, which resulted in a trajectory group with starting SBP values between 120 and 129 mmHg. For instance, in the normal-stable trajectory group, 54% of the participants had an optimal baseline BP, while 46% had a high-normal baseline BP (Additional file 1: Table S4). As a result, the trajectory for the normal-stable group began at an SBP of 123 mmHg (Fig. 2).

### Baseline BP groups and mortality risk

**Fig. 3:**
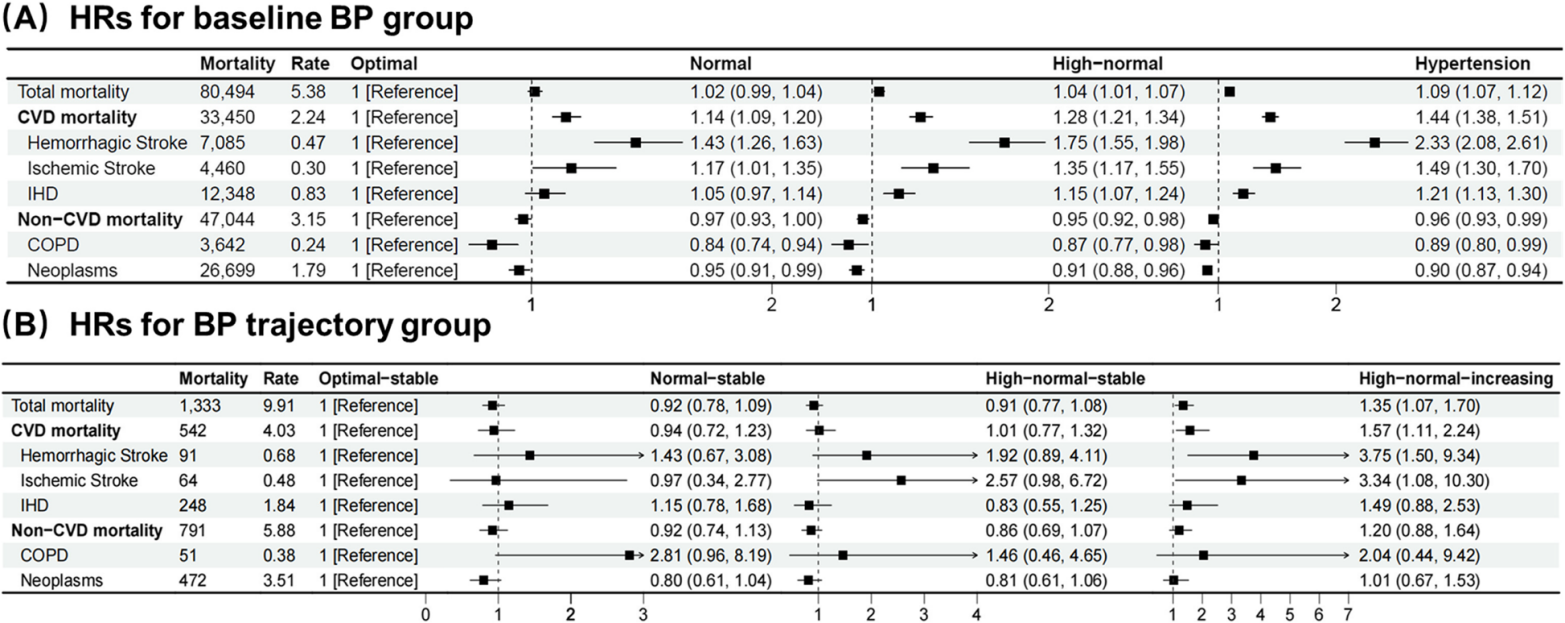
Multivariable adjusted hazard ratios for all-cause and cause-specific mortality in BP group and BP trajectory during the follow-up. Abbreviations: BP, blood pressure; SBP, systolic blood pressure; DBP, diastolic blood pressure; BMI, body mass index; HDL, high-density lipoprotein; LDL, low-density lipoprotein; TC, total cholesterol; TG, triglyceride; CVD, cardiovascular disease; IHD, ischemic heart disease; COPD, chronic obstructive pulmonary disease; HRs: hazard ratios. Rates were calculated as per 1,000 person-years. HRs were estimated after adjusted for sex, age, region, education level, annual household income, urbanity, smoking, alcohol consumption, BMI, TC, HDL, LDL, TG, fasting blood glucose, medical history, dyslipidemia, anti-hypertensive drug use and high CVD risk. BP group and BP trajectory pattern grouping criteria are the same as the footnote in Figs. 1 and 2

In the baseline BP analysis, the median follow-up duration was 4.5 years. There were 80,494 deaths during the follow-up period, corresponding to a crude mortality rate of 5.38 per 1,000 person-years, of which 33,450 deaths (2.24 per 1,000 person-years) resulted from CVD and 47,044 (3.15 per 1,000 person-years) resulted from non-CVD (Fig. 3A). Mortality for the different BP groups and subgroups is shown in the supplement (Additional file 1: Tables S5–S7).

Compared with the optimal BP group, individuals classified as having a high-normal BP exhibited a 4% higher risk of all-cause mortality (HR 1.04, 95% CI 1.02–1.08) following adjustment for other relevant variables. Additionally, this group showed a 28% elevated risk of CVD mortality (HR 1.28, 95% CI 1.21–1.34), with the most significant increase observed for hemorrhagic stroke mortality at 75% (HR 1.75, 95% CI 1.55–1.98). Individuals with normal BP (120–129/80–84 mmHg) had a 14% (HR 1.14, 95% CI 1.09–1.20) higher risk of CVD mortality with the most significant increase observed for hemorrhagic stroke mortality (HR 1.43, 95% CI 1.26–1.63).

In the subgroup analysis, the association between the BP groups and all-cause mortality was heterogeneous between age and sex subgroups (all P_interaction_ < 0.001). Women in the high-normal BP group exhibited a 6% increase in the risk of all-cause mortality (HR 1.06, 95% CI 1.01–1.11) compared with the optimal BP group, while the increase for men was not statistically significant. In the age subgroup analysis, individuals aged 35–44 and 45–54 years in the high-normal BP group had a 17% (HR 1.17, 95% CI 1.02–1.33) and 10% (HR 1.10, 95% CI 1.03–1.17) increase in all-cause mortality risk compared with the optimal BP group, respectively. However, for those aged 55–64 and 65–75 years, the increase in all-cause mortality risk among the high-normal BP group was not statistically significant (Additional file 1: Table S8, Table S9).

Compared with the primary analysis, there was no significant change in the relationship between baseline BP group and mortality after excluding deaths from the previous 3 years of follow-up or after further adjusting for lifestyle characteristics, such as dietary patterns and physical activity (Additional file 1: Table S10, Table S11).

### BP trajectory pattern during the follow-up and mortality risk

In the BP trajectory pattern analysis, the median follow-up time was 1.7 years. There were 1,333 deaths during the follow-up period, corresponding to a crude mortality rate of 9.91 per 1,000 person-years, with 542 deaths (4.03 per 1,000 person-years) resulting from CVD and 791 deaths (5.88 per 1,000 person-years) resulting from non-CVD (Fig. 3B). Mortality for different BP trajectory patterns and subgroups is shown in the supplement (Additional file 1: Tables S12–S14).

In contrast to the optimal-stable BP group, participants in the high-normal-increasing BP group had a 35% higher risk of all-cause mortality (HR 1.35, 95% CI 1.07–1.70) and a 57% higher risk of CVD mortality (HR 1.57, 95% CI 1.11–2.24), with the greatest increase for hemorrhagic stroke mortality (HR 3.75, 95% CI 1.50–9.34). There was no evidence of an association between the high-normal-increasing BP group and non-CVD mortality risk (HR 1.20, 95% CI 0.88–1.64).

In the subgroup analysis, no significant difference in the relationship was observed between BP trajectory patterns and all-cause mortality based on age and sex (all P_interaction_ > 0.05; Additional file 1: Table S15, Table S16).

### Attributable burden

**Fig. 4:**
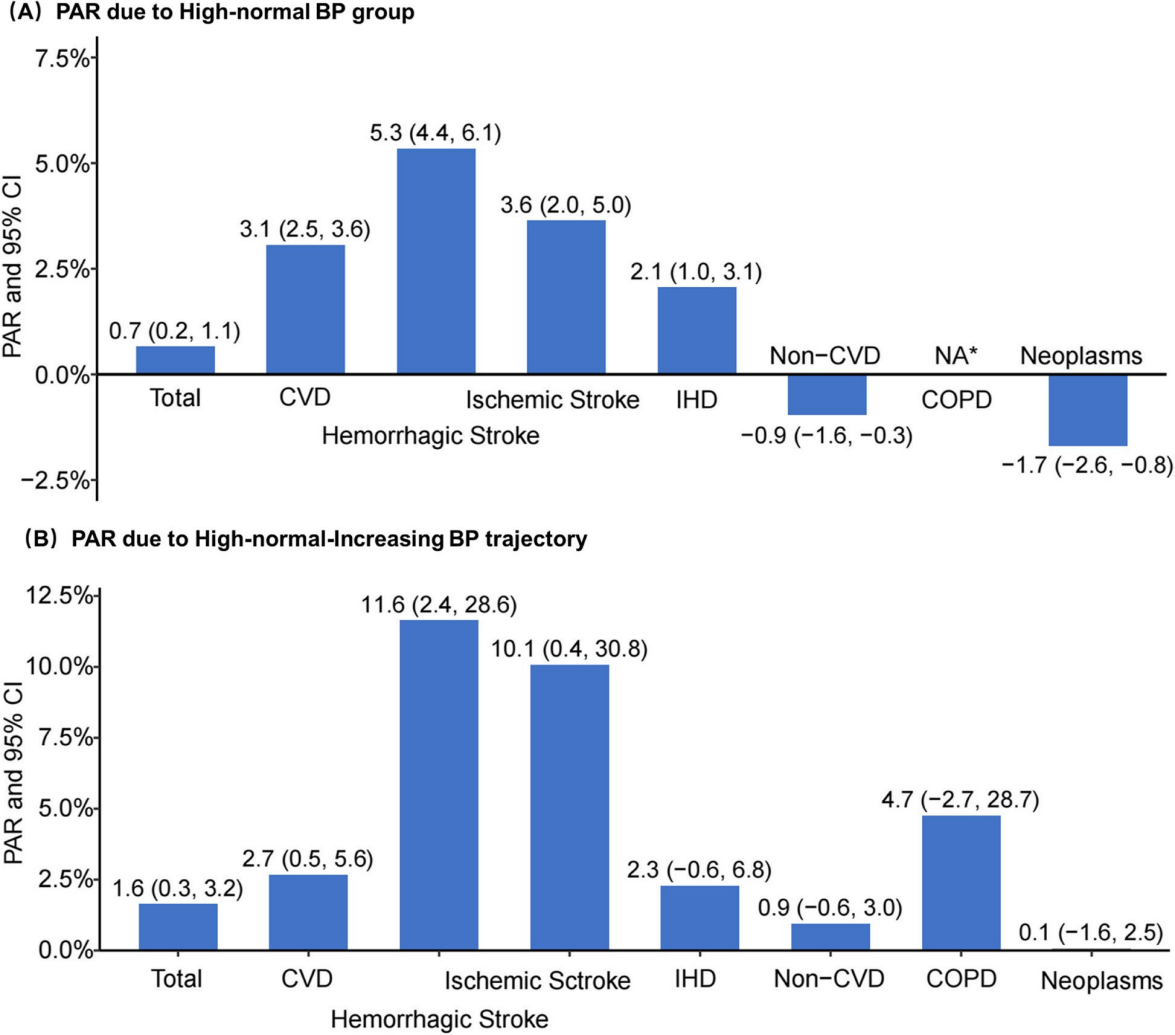
Population attributable risk for high-normal BP group and high-normal-increasing BP trajectory pattern. Abbreviations: PAR, population attributable risk; BP, blood pressure; CVD, cardiovascular disease. IHD, ischemic heart disease; COPD, chronic obstructive pulmonary disease. *NA represents the PAR value for COPD is too small to be estimated

Figure 4 shows that approximately 0.7% (95% CI: 0.2%–1.1%) and 1.6% (95% CI: 0.3%–3.2%) of all-cause mortality were attributable to high-normal baseline BP and the high-normal-increasing BP trajectory pattern, respectively. For CVD mortality, 3.1% (95% CI: 2.5%–3.6%) of mortality was attributable to high-normal baseline BP and 2.7% (95% CI: 0.5%–5.6%) of mortality was attributable to the high-normal-increasing trajectory pattern. The PAR for hemorrhagic stroke mortality was highest in the high-normal baseline BP group (5.3% [95% CI: 4.4%–56.1%]) and in the high-normal-increasing trajectory pattern group (11.6% [95% CI: 2.4%–28.6%]). Similar findings were observed in the subgroup analyses stratified by age and sex (Additional file 1: Figure S2, Figure S3).

## Discussion

This study found that both high-normal BP according to the definition of the ESH and the high-normal-increasing BP trajectory pattern during follow-up were associated with a significantly increased risk of all-cause mortality and CVD mortality, with the greatest increase observed for hemorrhagic stroke mortality. Among adults aged 35–54 years, and among women, a greater risk of mortality related to high-normal baseline BP was observed.

This study, based on a large cohort, extends the literature in several ways. First, in addition to the increased risk of all-cause and CVD mortality, this study observed that high-normal BP at baseline was related to a particularly pronounced risk of hemorrhagic stroke mortality. Several biological mechanisms could explain this finding. Cerebral small vessel disease is more common in patients with high-normal BP and is an important factor contributing to hemorrhagic stroke. High-normal BP may exacerbate chronic damage to the vessel wall, particularly affecting the integrity of small arteries and leading to lipid vitreous degeneration [24]. This increases the risk of rupture under stressful conditions, thereby predisposing individuals to hemorrhagic stroke [25]. The present study identified that 5.3% of hemorrhagic stroke mortality was attributed to high-normal BP. Similar results were found in the BP trajectory group. Similarly, Zhao et al. found that 12.4% of stroke mortality was attributable to the 130–139/80–89 mmHg BP range [26]. Even though the PAR calculation was not based on a representative sample of the entire population, the findings provide more cause-specific insights into the health impact of high-normal BP.

In our study, baseline high-normal BP (130–139/85–89 mmHg) was associated with an increased risk of all-cause mortality and CVD mortality. Further trajectory analysis revealed significant heterogeneity. Individuals with a high-normal-increasing pattern, rather than those with a high-normal-stable trajectory, exhibited significantly elevated mortality risks. These findings suggest that the association between baseline high-normal BP and mortality may be primarily driven by the subset of individuals who experienced a progressive BP elevation over time. Emerging evidence have shown that long-term BP elevation harms cardiovascular health. For instance, a study based on 21,441 participants from the Chinese multi-provincial cohort study found that individuals with a baseline BP in the range of 130–139/80–89 mmHg and a BP ≥ 140/≥ 90 mmHg at the last follow-up had a three-fold higher risk of CVD than individuals whose BP consistently remained below 130/80 mmHg [26]. The Kailuan cohort, which involved 79,385 community-dwelling adults, found that individuals with long-term SBP ranging from 120 to 140 mmHg had a two-fold higher risk of stroke than those whose SBP consistently remained below 120 mmHg [20].

The following mechanisms may explain the results of this study. Even without reaching the diagnostic criteria for hypertension, long-term BP elevation could lead to damage and inflammation of the arterial walls, promoting the occurrence of atherosclerosis [27]. Atherosclerosis could cause blood vessel narrowing and decreased elasticity, increasing the burden on the heart, thus increasing CVD risk [28]. In addition, long-term BP elevation often accompanies other metabolic issues, such as obesity, diabetes mellitus, and high cholesterol [29]. These factors collectively increase mortality risk.

The findings of this study also showed heterogeneity in the association between high-normal BP at baseline and mortality across different age and sex subgroups. Specifically, adults aged 35–54 years and women exhibited an increased likelihood of experiencing an elevated mortality risk. A meta-analysis involving a total of 250,000 individuals revealed that the occurrence of high-normal BP was notably greater in women than in men [30]. Other epidemiological studies have also reported higher CVD mortality in women with high-normal BP compared to men, which is consistent with our findings [31–33]. The physiological structure and function of the cardiovascular system may explain the sex differences in mortality risk. For instance, sympathetic nerve activity is usually more active in women than in men [34]. Women's arteries are thinner than men's arteries, which puts relatively more pressure on the arteries and makes women more susceptible to CVD at high-normal BP levels [35]. Furthermore, woman sex hormones, such as estrogen, exert a protective effect during the premenopausal period, reducing the incidence of CVD. However, postmenopausal estrogen levels decline, resulting in a significant increase in CVD risk among women [36]. Notably, there was a significant increase in mortality among individuals aged 35–54 years, whereas there was no significant increase in people aged ≥ 55 years. The vascular system of middle-aged adults is often in a transitional phase of increased stiffness and accumulation of atherosclerotic changes. Elevated BP in the high-normal range may exacerbate the rate of these vascular changes and accelerate progression to clinically significant CVD. Previous longitudinal studies have confirmed that midlife BP is a predictor of late-life CVD events, supporting the notion that high-normal BP may have a greater impact during this critical period [18, 37].

This study showed that individuals within normal BP range (120–129/80–84 mmHg) also had an increased risk of CVD mortality, particularly hemorrhagic stroke mortality. The elevated CVD risk in this group may be attributed to subtle, undetected vascular dysfunction or metabolic disturbances that predispose individuals to CVD events [38]. Importantly, these results underscore the need for a broader understanding of BP since individuals within the normal range can benefit from targeted surveillance and behavioral intervention. Further research is necessary, as our study relied on baseline BP measurements and did not account for other more precise assessments such as ambulatory BP monitoring that can help identify masked hypertension among those individuals within normal BP range[39].

The findings of the present study have potential implications for BP management. The prevalence of high-normal BP (130–139/85–89 mmHg) was 18.7% in the present study. Extrapolating these results, it is estimated that 137 million patients aged between 35 and 75 years in China have high-normal BP, and 77 million of these individuals will develop hypertension within 5 years. Therefore, early management of BP in patients with high-normal BP is crucial for the prevention and treatment of hypertension. Therefore, ensuring early detection of high-normal BP is important. However, the awareness rate of hypertension in China is < 50%, and the awareness rate of high-normal BP is even lower [40, 41]. Community health services play a significant role in early detection by performing regular BP monitoring [42]. Residents should be encouraged to regularly participate in these health check-ups, and facilities should provide convenient times and locations to increase participation. Basic public health services in residential communities should also be enhanced by establishing health records for BP monitoring, along with conducting regular follow-up visits [43, 44]. Health education activities should be actively organized to provide relevant knowledge about high-normal BP. Additionally, individuals with high-normal BP require more intensive intervention. Previous studies have shown that reducing salt intake [45], increasing moderate-intensity aerobic exercise [46], and maintaining an appropriate body weight [47] can effectively reduce the likelihood of these individuals developing hypertension. Moreover, pharmacologic interventions may be considered for patients with high-normal BP. Jingtang et al. demonstrated that antihypertensive pharmacologic therapy in the 130–139/80–89 mmHg BP range offers significant cost-effectiveness, with the greatest benefits observed among younger and middle-aged individuals [3].

### Study limitations

This study has several limitations. First, collider bias may have existed when exploring the association between BP and mortality. To address this bias, sensitivity analysis was performed after excluding deaths within 3 years of follow-up, and the results were consistent with the primary analysis. Second, although we adjusted potential confounding factors as thoroughly as possible, we cannot completely rule out the impact of other unknown confounding factors on the results. To assess this potential impact, we additionally adjusted for diet and physical activity, two important confounding factors, in the sensitivity analysis. The analysis results showed that even after adjusting for these variables, there was no substantial change in the estimated effect of the model, indicating a low likelihood of residual confounding. Therefore, we believe that the current research results are stable, robust, and reliable. Finally, recall bias and measurement error were inevitable due to the reliance on self-reported data for certain variables, such as disease history, although we used a standardized questionnaire that had been previously used and validated in other large population studies [48].

## Conclusions

High-normal BP (130–139/85–89 mmHg) at baseline and its trajectory pattern during follow-up were associated with an increased risk of all-cause mortality and CVD mortality. The positive association between baseline high-normal BP and mortality may mainly stem from the “high-normal-increasing” BP change over time, and these findings highlight the necessity of regular, targeted BP surveillance and behavioral intervention in managing hypertension and cardiovascular events.

## Supplementary Information

Additional file 1: Figure S1. Study sites in ChinaHEART; Figure S2. Population attributable risk due to"High-normal"in baseline BP groups; Figure S3. Population attributable risk due to"High normal-increasing"in BP trajectory patterns; Table S1. Number of participants with different BP measurements in baseline BP groups; Table S2. Number of participants with different BP measurements with trajectory patterns; Table S3. Characteristics of participants in ChinaHEART study with different BP trajectory patterns; Table S4. Number of participants in trajectory patterns and baseline BP groups; Table S5. Death casesin baseline BP groups; Table S6. Death casesin baseline BP groups by sex group; Table S7. Death casesin baseline BP groups by age group; Table S8. Multivariable adjusted hazard ratios for all-cause and cause-specific mortality in baseline BP groups by sex group; Table S9. Multivariable adjusted hazard ratios mortality in baseline BP groups by age group; Table S10. Comparison of results between the main model and the model after excluding the first 3 years of death in baseline BP groups; Table S11. Comparison of results between the main model and the model additionally adjustment for diet and physical activity in baseline BP groups; Table S12. Death casesin BP trajectory patterns; Table S13. Death casesin BP trajectory patterns by sex group; Table S14. Death casesin BP trajectory patterns by age group; Table S15. Multivariable adjusted hazard ratios for all-cause and cause-specific mortality in BP trajectory patterns by sex group; Table S16. Multivariable adjusted hazard ratios for all-cause and cause-specific mortality in BP trajectory patterns by age group. 

## Data Availability

The data are not publicly available. The China Health Evaluation And risk Reduction Through nationwide teamwork (ChinaHEART) project only provides conditional data access for qualified researchers with legitimate requests; a formal application and research proposal is required. Please contact cvd-project@nccd.org.cn to seek approval for data access.

## Abbreviations

BP: Blood pressure
BMI: Body mass index
ChinaHEART: China health evaluation and risk reduction through nationwide teamwork
COPD: Chronic obstructive pulmonary disease
CVD: Cardiovascular disease
DBP: Diastolic BP
ESH: European society of hypertension
HDL: High-density lipoprotein
HR: Hazard ratio
ICD- 10: International classification of diseases
IHD: Ischemic heart disease
LDL: Low-density lipoprotein
PAR: Population attributable risk
SBP: Systolic BP
