# Supplementary material for: Association of high-normal blood pressure defined by the 2023 European Society of Hypertension guideline with mortality in the Chinese population: a nationwide, population-based, prospective study of 3.6 million adults
Source: BMC Med. 2025 Apr 16;23:226. doi: 10.1186/s12916-025-04055-5 (PMC12004562; doi:10.1186/s12916-025-04055-5)
Supplement: Supplementary file 1 — Additional file 1: Figure S1. Study sites in ChinaHEART; Figure S2. Population attributable risk due to"High-normal"in baseline BP groups; Figure S3. Population attributable risk due to"High normal-increasing"in BP trajectory patterns; Table S1. Number of participants with different BP measurements in baseline BP groups; Table S2. Number of participants with different BP measurements with trajectory patterns; Table S3. Characteristics of participants in ChinaHEART study with different BP trajectory patterns; Table S4. Number of participants in trajectory patterns and baseline BP groups; Table S5. Death casesin baseline BP groups; Table S6. Death casesin baseline BP groups by sex group; Table S7. Death casesin baseline BP groups by age group; Table S8. Multivariable adjusted hazard ratios for all-cause and cause-specific mortality in baseline BP groups by sex group; Table S9. Multivariable adjusted hazard ratios mortality in baseline BP groups by age group; Table S10. Comparison of results between the main model and the model after excluding the first 3 years of death in baseline BP groups; Table S11. Comparison of results between the main model and the model additionally adjustment for diet and physical activity in baseline BP groups; Table S12. Death casesin BP trajectory patterns; Table S13. Death casesin BP trajectory patterns by sex group; Table S14. Death casesin BP trajectory patterns by age group; Table S15. Multivariable adjusted hazard ratios for all-cause and cause-specific mortality in BP trajectory patterns by sex group; Table S16. Multivariable adjusted hazard ratios for all-cause and cause-specific mortality in BP trajectory patterns by age group. [file 12916_2025_4055_MOESM1_ESM.docx]

**Association of high-normal blood pressure defined by the 2023 European Society of Hypertension guideline with mortality in the Chinese population: A nationwide, population-based, prospective study of 3.6 million adults**

Zhiwei Li^1#^, PhD; Mengmeng Liu^2#^, MSc; Bowang Chen^1^, PhD; Yuelin Wu^1^, MSc; Hui Jia^2^, MSc; Ruirui Geng^2^, MSc; Yixiao Wang^1^, BA; Xiaoyan Zhang^1^, MSc; Yang Yang^1^, PhD; Jianlan Cui^1^, MSc; Jiapeng Lu^1^, PhD; Zhiping Guo^2,3*^, PhD; Xi Li^1,2,4*^, PhD; Weili Zhang^1,2*^, PhD, MD

1. National Clinical Research Center of Cardiovascular Diseases, Fuwai Hospital, National Center for Cardiovascular Diseases, Chinese Academy of Medical Sciences and Peking Union Medical College, Beijing 100037, People’s Republic of China.
2. Central China Subcenter of National Center for Cardiovascular Diseases, Henan Cardiovascular Disease Center, Fuwai Central-China Cardiovascular Hospital, Central China Fuwai Hospital of Zhengzhou University, Zhengzhou 450000, People’s Republic of China.
3. Henan Key Laboratory of Chronic Disease, Fuwai Central China Cardiovascular Hospital, Zhengzhou 450000, People’s Republic of China
4. Shenzhen Clinical Research Center for Cardiovascular Diseases, Fuwai Hospital Chinese Academy of Medical Sciences, Shenzhen, Shenzhen 518000, People’s Republic of China.

# These authors contributed equally to this work

* Corresponding author

**Correspondence to:**

Weili Zhang, PhD, National Clinical Research Center for Cardiovascular Diseases, Fuwai Hospital, 167 Beilishi Road, Beijing 100037, People’s Republic of China;

Email: zhangweili@fuwaihospital.org

Xi Li, PhD, National Clinical Research Center for Cardiovascular Diseases, Fuwai Hospital, 167 Beilishi Road, Beijing 100037, People’s Republic of China;

Email: xi.li@nccd.org.cn

Zhiping Guo, PhD, .Henan Key Laboratory of Chronic Disease, Fuwai Central China Cardiovascular Hospital, Central China Subcenter of National Center for Cardiovascular Diseases, Henan Cardiovascular Disease Center, Fuwai Central-China Cardiovascular Hospital, Central China Fuwai Hospital of Zhengzhou University, Zhengzhou 450000, People’s Republic of China.;

Email: zpguo@zzu.edu.cn

**Supplementary files**

**Figure count**: 3

**Table count**: 16

[Figure S1. Study sites in ChinaHEART 4](#_Toc185160436)

[Table S1. Number of participants with different BP measurements in baseline BP groups during the follow-up 8](#_Toc185160437)

[Table S2. Number of participants with different BP measurements with trajectory pattern groups during the follow-up 9](#_Toc185160438)

[Table S3. Characteristics of participants in ChinaHEART study with different blood pressure trajectory pattern during the follow-up 10](#_Toc185160439)

[Table S4. Number of participants in trajectory patterns and baseline BP groups 13](#_Toc185160440)

[Table S5. Death cases (%) for all-cause and cause-specific disease in baseline BP group according to 2023 ESH Guidelines 14](#_Toc185160441)

[Table S6. Death cases (%) for all-cause and cause-specific disease in baseline BP group according to 2023 ESH Guidelines by sex 15](#_Toc185160442)

[Table S7. Death cases (%) for all-cause and cause-specific disease in baseline BP group according to 2023 ESH Guidelines by age group 16](#_Toc185160443)

[Table S8. Multivariable adjusted hazard ratios for all-cause and cause-specific mortality in baseline BP group according to 2023 ESH Guidelines by sex 18](#_Toc185160445)

[Table S9. Multivariable adjusted hazard ratios for all-cause and cause-specific mortality in baseline BP group according to 2023 ESH Guidelines by age group 19](#_Toc185160446)

[Table S10. Comparison of results between the main model and the model after excluding the first 3 years of death (New model) in baseline BP group 21](#_Toc185160447)

[Table S11. Comparison of results between the main model and the model additionally adjustment for diet and physical activity (New model) in baseline BP group 23](#_Toc185160448)

[Table S12. Death cases (%) for all-cause and cause-specific disease in BP trajectory pattern during the follow-up 25](#_Toc185160449)

[Table S13. Death cases (%) for all-cause and cause-specific disease in BP trajectory pattern during the follow-up by sex 26](#_Toc185160450)

[Table S14. Death cases (%) for all-cause and cause-specific disease in BP trajectory pattern during the follow-up by age group 27](#_Toc185160451)

[Table S15. Multivariable adjusted hazard ratios for all-cause and cause-specific mortality in BP trajectory pattern during the follow-up by sex 29](#_Toc185160452)

[Table S16. Multivariable adjusted hazard ratios for all-cause and cause-specific mortality in BP trajectory pattern during the follow-up by age group 30](#_Toc185160453)

[Figure S2. Population attributable risk (PAR) due to "High-normal" in baseline BP group by sex and age group 32](#_Toc185160454)

[Figure S3. Population attributable risk (PAR) due to "High normal-Increasing" in BP trajectory pattern during the follow-up by sex and age group 33](#_Toc185160455)

**The criteria for selecting study sites and populations for the China Health Evaluation And risk Reduction Through nationwide teamwork (ChinaHEART) project**

**1) Study sites**

From November 2014 to December 2022, a total of 359 sites across all 31 provinces were carefully chosen based on their geographical locations within each province, the population residing in rural and urban areas, the distribution of minority ethnicities, the quality of disease and death registries, as well as the local capacity to support the project. The provincial coordinating office staff played a crucial role in gathering essential data regarding the selected sites in their respective provinces, including geographic information, economic development, population size, and distribution of minority ethnicities. This information was then shared with the national coordinating office, where it was thoroughly reviewed and discussed with the staff to determine the final study sites. Each site comprised approximately 8-9 towns or sub-districts, selected based on factors such as population size, population stability (ensuring no sudden significant changes), the commitment of local staff, and their ability to conduct the required screening. Initial screening stations were established within the health centers of each town or sub-district.


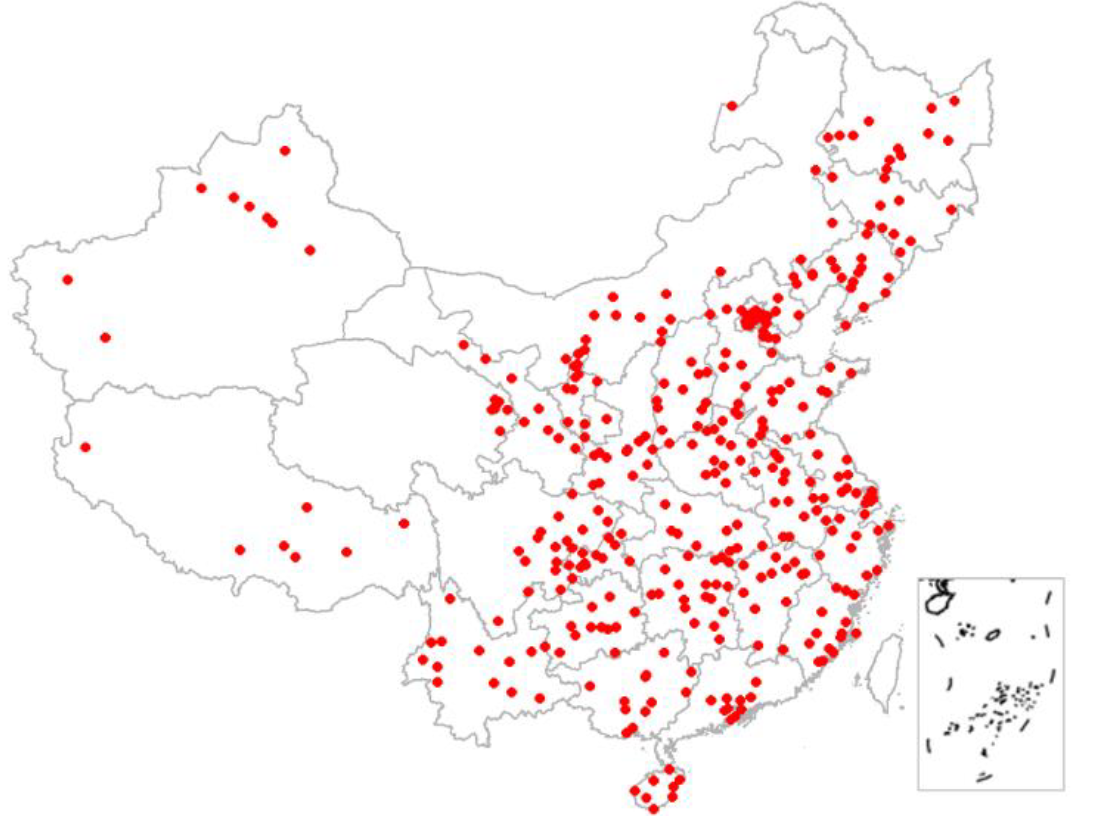


**Figure S1. Study sites in ChinaHEART**

**2) Participants recruitment**

In every town or sub-district, potentially eligible participants were identified by referring to official residential records. Subsequently, local community workers extended invitations to these individuals through telephone communications or extensive publicity campaigns on television and in newspapers. Each participant was required to present their identity cards at the screening clinics to ensure compliance with the inclusion criteria: 1) age between 35 and 75 years; 2) registration in the selected site's Hukou (an official record confirming residency in a specific area) and having resided in the selected regions for at least 6 months within the past 12 months. Upon verification, eligible participants who had willingly signed the informed consent agreement were successfully enrolled in the project.

For each site, a predetermined annual target number of screenings was established based on the population size. Between November 2014 and December 2022, a total of 12,323,531 local residents aged 35-75 years received invitations, resulting in the recruitment of 4.7 million participants into the project. The overall response rate was 36.5%.

**3) Definition of high CVD risk**

High CVD risk should meet at least one of four criteria: (i) major cardiovascular events history (myocardial infarction, percutaneous coronary intervention, coronary artery bypass graft or stroke); (ii) a predicted CVD risk ≥ 20% based on World Health Organization(WHO)/International Society of Hypertension cardiovascular risk prediction charts; (iii) severely abnormal blood lipid levels (LDL ≥ 4.14 mmol/L or HDL <0.78 mmol/L); or (iv) severely high BP (SBP >160 mmHg or DBP >100 mmHg).

**4) Physical activity**

We measured habitual exercise by asking typical type of activity at different intensity (vigorous or moderate), frequency, and exercise time per week.

**Typical type and intensity of physical activity**

| **Type** | **Intensity*** |
| --- | --- |
| Tai-Chi/qigong/leisure walking | Moderate |
| Running/aerobic exercise | Vigorous |
| Ball games (e.g., basketball, table tennis, badminton) | Moderate |
| Brisk walking/gymnastics/folk dancing | Moderate |
| Swimming | Vigorous |
| Other exercise (e.g., mountain walking, home exercise and rope jumping) | Moderate |

* The intensity for each type was adopted in a prior study: Zhu N, Yu C, Guo Y, et al. Adherence to a healthy lifestyle and all cause and cause-specific mortality in Chinese adults: a 10-year prospective study of 0.5 million people. International Journal of Behavioral Nutrition and Physical Activity 2019;16(1):98.

**5) Assessment of food intake**

Habitual food intake frequency during the past year was asked in the questionnaire: ‘daily’, ‘4-6 days per week’, ‘1-3 days per week’, ‘1-3 days per month’, ‘never or almost never’. In this study we focused on 6 food groups: fresh fruit, fresh vegetable, whole grains (mainly referring to crops except rice and flour, including millet, corn, sorghum, sweet potato, etc.), fish and other seafood (referring to fish, shrimp, crab, shellfish, snails, etc.), bean and bean products (referring to all kinds of soy foods including tofu, dried tofu, beverage made from soybeans, etc.), and red meat (referring to a variety of fresh or processed meat such as pork, beef, lamb, etc.).

Healthy diet was defined as a combination of 6 dietary scores greater than or equal to 4.

**The definition of healthy diet score**

| **Food groups** | **‘Healthy’ (score = 1)** |
| --- | --- |
| Fresh fruit | every day per week |
| Fresh vegetables | every day per week |
| Whole grains | every day per week |
| Fish and other seafood | ≥1 day per week |
| Bean and bean products | ≥4 days per week |
| Red meat | <7 days per month |

**Table S1. Number of participants with different BP measurements in baseline BP groups during the follow-up**

| **BP measurements** | **Baseline BP groups** | | | | **Overall**  **(N = 3,598,940)** |
| --- | --- | --- | --- | --- | --- |
|  | **Optimal**  **(N = 652,400)** | **Normal**  **(N = 700,948)** | **High-normal**  **(N = 674,509)** | **Hypertension**  **(N = 1,571,083)** |  |
| 1 | 597,188 | 643,168 | 613,680 | 986,460 | 2,840,496 |
| 2 | 15,951 | 15,996 | 16,472 | 140,756 | 189,175 |
| 3 | 12,147 | 12,396 | 12,750 | 125,361 | 162,654 |
| 4 | 9,207 | 9,646 | 10,076 | 101,434 | 130,363 |
| 5 | 7,181 | 7,589 | 8,023 | 82,603 | 105,396 |
| 6 | 5,153 | 5,410 | 5,736 | 58,237 | 74,536 |
| 7 | 3,393 | 4,025 | 4,496 | 44,581 | 56,495 |
| 8 | 2,002 | 2,425 | 2,836 | 27,643 | 34,906 |
| 9 | 178 | 293 | 440 | 4,008 | 4,919 |
| Overall | 652,400 | 700,948 | 674,509 | 1,571,083 | 3,598,940 |

Abbreviations: BP, blood pressure.

**Table S2. Number of participants with different BP measurements with trajectory pattern groups during the follow-up**

| **BP measurements** | **Trajectory pattern groups** | | | | **Overall**  **(N =78,130)** |
| --- | --- | --- | --- | --- | --- |
|  | **Optimal-stable (N = 20,419)** | **Normal-stable (N =30,606)** | **High-normal-stable (N = 23,363)** | **High-normal-increasing (N =3,742)** |  |
| 3 | 6091 | 8954 | 7034 | 1128 | 23207 |
| 4 | 4678 | 6987 | 5457 | 909 | 18031 |
| 5 | 3724 | 5623 | 4250 | 646 | 14243 |
| 6 | 2750 | 4071 | 2879 | 473 | 10173 |
| 7 | 1909 | 2998 | 2142 | 309 | 7358 |
| 8 | 1150 | 1737 | 1395 | 251 | 4533 |
| 9 | 117 | 236 | 206 | 26 | 585 |
| Overall | 20419 | 30606 | 23363 | 3742 | 78130 |

Abbreviations: BP, blood pressure.

**Table S3. Characteristics of participants in ChinaHEART study with different blood pressure trajectory pattern during the follow-up**

| **Characteristics** | **Overall (N =78,130)** | **Optimal-stable (N = 20,419)** | **Normal-stable (N =30,606)** | **High-normal-stable (N = 23,363)** | **High-normal-increasing (N =3,742)** |
| --- | --- | --- | --- | --- | --- |
| SBP, mm Hg, mean (SD) | 122.80 (12.5) | 109.78(7.6) | 122.93 (10.9) | 132.37 (7.2) | 133.02 (7.2) |
| DBP, mm Hg, mean (SD) | 75.06 (7.2) | 69.95 (5.2) | 75.14 (6.8) | 78.82 (6.7) | 78.83 (6.7) |
| Men, n (%) | 29,866 (38.2) | 6,408 (31.4) | 12,173 (39.8) | 9,851 (42.2) | 1,434 (38.3) |
| Age, year, n (%) | 54.94 (9.5) | 50.90 (9.0) | 54.52 (9.3) | 58.05 (8.9) | 60.96 (8.4) |
| 35-44 | 12,504 (16.0) | 5,545 (27.2) | 4951 (16.2) | 1850 (7.9) | 158 (4.2) |
| 45-54 | 25,735 (32.9) | 8,163 (40.0) | 10,603 (34.6) | 6,283 (26.9) | 686 (18.3) |
| 55-64 | 25,554 (32.7) | 4,975 (24.4) | 10,064 (32.9) | 9,075 (38.8) | 1,440 (38.5) |
| 65-75 | 14,337 (18.4) | 1,736 (8.5) | 4,988 (16.3) | 6,155 (26.3) | 1,458 (39.0) |
| Region, n (%) |  |  |  |  |  |
| East China | 25,746 (33.0) | 6,115 (29.9) | 9,771 (31.9) | 8,315 (35.6) | 1,545 (41.3) |
| Central China | 18,998 (24.3) | 4,925 (24.1) | 7,358 (24.0) | 5,849 (25.0) | 866 (23.1) |
| West China | 28,048 (35.9) | 8,224 (40.3) | 11,381 (37.2) | 7,449 (31.9) | 994 (26.6) |
| Northeast China | 5,338 (6.8) | 1,155 (5.7) | 2,096 (6.8) | 1,750 (7.5) | 337 (9.0) |
| Education, n (%) |  |  |  |  |  |
| Primary school and below | 28,870 (37.0) | 5,539 (27.1) | 11,156 (36.5) | 10,175 (43.6) | 2,000 (53.4) |
| middle school | 25,090 (32.1) | 6,447 (31.6) | 10,038 (32.8) | 7,541 (32.3) | 1,064 (28.4) |
| High school and above | 23,223 (29.7) | 8,254 (40.4) | 8,997 (29.4) | 5,357 (22.9) | 615 (16.4) |
| Unknown | 947 (1.2) | 179 (0.9) | 415 (1.4) | 290 (1.2) | 63 (1.7) |
| Annual household income, RMB, n (%) |  |  |  |  |  |
| <10k | 11,880 (15.2) | 2,439 (11.9) | 4,508 (14.7) | 4,120 (17.6) | 813 (21.7) |
| 10k-50k | 42,325 (54.2) | 10,718 (52.5) | 16,563 (54.1) | 12,961 (55.5) | 2,083 (55.7) |
| >=50k | 17,005 (21.8) | 5,517 (27.0) | 6,754 (22.1) | 4,266 (18.3) | 468 (12.5) |
| Unknown | 6,920 (8.9) | 1,745 (8.5) | 2,781 (9.1) | 2,016 (8.6) | 378 (10.1) |
| Live in Rural, n (%) | 44,560 (57.0) | 11,036 (54.0) | 17,200 (56.2) | 13,761 (58.9) | 2,563 (68.5) |
| Current smoker, n (%) | 16,516 (21.1) | 3,962 (19.4) | 6,740 (22.0) | 5,105 (21.9) | 709 (18.9) |
| Current drinker, n (%) | 19,716 (25.2) | 5,015 (24.6) | 7,840 (25.6) | 6,002 (25.7) | 859 (23.0) |
| BMI, kg/m^2^, n (%) | 24.41 (3.2) | 23.39 (2.9) | 24.45 (3.1) | 25.12 (3.2) | 25.22 (3.4) |
| <18 | 860 (1.1) | 386 (1.9) | 283 (0.9) | 158 (0.7) | 33 (0.9) |
| 18-24 | 46,125(59.1) | 14,550 (71.3) | 18,009 (58.9) | 11,747 (50.3) | 1,819 (48.7) |
| 24-28 | 21,064 (27.0) | 4,125 (20.2) | 8,429 (27.6) | 7,355 (31.5) | 1,155 (30.9) |
| >=28 | 9,987 (12.8) | 1,339 (6.6) | 3,840 (12.6) | 4,077 (17.5) | 731 (19.6) |
| Total cholesterol, mmol/L, n (%) | 5.07 (1.5) | 4.90 (1.4) | 5.04 (1.5) | 5.20 (1.5) | 5.35 (1.5) |
| <5.2 | 46,306 (59.3) | 13,124 (64.3) | 18,357 (60.0) | 12,958 (55.5) | 1,867 (49.9) |
| 5.2-6.19 | 11,214 (14.4) | 2,816 (13.8) | 4,304 (14.1) | 3,505 (15.0) | 589 (15.7) |
| >=6.2 | 20,610 (26.4) | 4,479 (21.9) | 7,945 (26.0) | 6,900 (29.5) | 1,286 (34.4) |
| HDL-cholesterol, mmol/L, mean (SD) | 1.37 (0.5) | 1.40 (0.5) | 1.36 (0.5) | 1.36 (0.5) | 1.39 (0.4) |
| LDL-cholesterol, mmol/L, mean (SD) | 2.97 (1.3) | 2.82 (1.3) | 2.95 (1.3) | 3.08 (1.4) | 3.21 (1.4) |
| Triglycerides, mmol/L, median (IQR) | 1.62 (0.9) | 1.49 (0.8) | 1.65 (0.9) | 1.70 (0.9) | 1.68 (0.9) |
| Fasting blood glucose, mmol/L, n (%) | 6.18 (1.7) | 5.91 (1.4) | 6.17 (1.7) | 6.34 (1.9) | 6.58 (2.2) |
| <5.6 | 28,835 (36.9) | 8,900 (43.6) | 11,141 (36.4) | 7,660 (32.8) | 1,134 (30.3) |
| 5.6-6.9 | 36,817 (47.1) | 9,391 (46.0) | 14,571 (47.6) | 11,113 (47.6) | 1,742 (46.6) |
| >=7.0 | 12,478 (16.0) | 2,128 (10.4) | 4,894 (16.0) | 4,590 (19.6) | 866 (23.1) |
| Medical history, n (%) |  |  |  |  |  |
| Cardiovascular disease | 13,007 (16.6) | 2,583 (12.6) | 4,917 (16.1) | 4680 (20.0) | 827 (22.1) |
| Stroke | 7,710 (9.9) | 1,392 (6.8) | 2,890 (9.4) | 2902 (12.4) | 526 (14.1) |
| Myocardial infarction | 3,321 (4.3) | 761 (3.7) | 1,286 (4.2) | 1080 (4.6) | 194 (5.2) |
| Hypertension | 0 (0.0) | 0 (0.0) | 0 (0.0) | 0 (0.0) | 0 (0.0) |
| Diabetes mellitus | 5,266 (6.7) | 865 (4.2) | 2,057 (6.7) | 1,941 (8.3) | 403 (10.8) |
| Dyslipidemia, n (%) | 26,462 (33.9) | 5,690 (27.9) | 10,257 (33.5) | 8,901 (38.1) | 1,614 (43.1) |
| Anti-hypertensive drug use, n(%) | 0 (0.0) | 0 (0.0) | 0 (0.0) | 0 (0.0) | 0 (0.0) |
| High CVD risk ^a^, n (%) | 45,346 (58.0) | 9,938 (48.7) | 17,751 (58.0) | 15,056 (64.4) | 2,601 (69.5) |
| Mortality, n (%) |  |  |  |  |  |
| Total mortality | 1,333 (1.7) | 256 (1.3) | 503 (1.6) | 452 (1.9) | 122 (3.3) |
| CVD mortality | 542 (0.7) | 96 (0.5) | 190 (0.6) | 196 (0.8) | 60 (1.6) |
| Non-CVD mortality | 91 (0.1) | 10 (0.0) | 30 (0.1) | 37 (0.2) | 14 (0.4) |

Abbreviations: BP, blood pressure; SD, standard deviation; SBP, systolic blood pressure; DBP, diastolic blood pressure; BMI, body mass index (calculated as weight in kilograms divided by height in meters squared); HDL, High-Density Lipoprotein; LDL, Low-Density Lipoprotein; IQR, interquartile range; CVD, Cardiovascular Disease.

SI conversion factors: To convert HDL, LDL and total cholesterol to mg/dL, divide by 0.0259; Fasting blood glucose to mg/dL, divide by 0.0555; Triglycerides to mg/dL, divide by 0.0113.

^a^: High CVD risk should meet at least one of four criteria: (i) major cardiovascular events history (myocardial infarction, percutaneous coronary intervention, coronary artery bypass graft or stroke); (ii) a predicted CVD risk ≥ 20% based on World Health Organization(WHO)/International Society of Hypertension cardiovascular risk prediction charts; (iii) severely abnormal blood lipid levels (LDL ≥ 4.14 mmol/L or HDL <0.78 mmol/L); or (iv) severely high blood pressure (SBP >160 mmHg or DBP >100 mmHg).

Blood pressure trajectory pattern group reference Figure 2.

**Table S4. Number of participants in trajectory patterns and baseline BP groups**

| **Trajectory**  **patterns** | **Baseline BP groups [N (%)]** | | **Overall** |
| --- | --- | --- | --- |
|  | **Optimal** | **High-normal** |  |
| Optimal-stable | 19,706 (97%) | 713 (3%) | 20,419 |
| Normal-stable | 16,444 (54%) | 14,162 (46%) | 30,606 |
| High-normal-stable | 2,743 (12%) | 20,620 (88%) | 23,363 |
| High-normal-increasing | 368 (10%) | 3,374 (90%) | 3,742 |
| Overall | 39,261 (50%) | 38,869 (50%) | 78,130 |

Optimal: SBP < 120 mmHg and DBP < 80 mmHg; High-normal: SBP:130-139 mmHg and/or DBP: 85-89 mmHg

**Table S5. Death cases (%) for all-cause and cause-specific** **mortality in baseline BP group according to 2023 ESH guideline**

|  | **Optimal (N = 652,400)** | **Normal (N = 700,948)** | **High-normal (N = 674,509)** | **Hypertension (N = 1,571,083)** |
| --- | --- | --- | --- | --- |
| Systolic/diastolic BP, mm Hg | <120/<80 | 120-129/80-84 | 130-139/85-89 | >=140/>=90 |
| Total mortality | 8611 (1.3) | 11265 (1.6) | 13092 (1.9) | 47526 (3.0) |
| CVD mortality | 2492 (0.4) | 3725 (0.5) | 4741 (0.7) | 22492 (1.4) |
| Hemorrhagic Stroke | 381 (0.1) | 666 (0.1) | 884 (0.1) | 5154 (0.3) |
| Ischemic Stroke | 290 (0.0) | 463 (0.1) | 633 (0.1) | 3074 (0.2) |
| IHD | 1070 (0.2) | 1521 (0.2) | 1931 (0.3) | 7826 (0.5) |
| Non CVD mortality | 6119 (0.9) | 7540 (1.1) | 8351 (1.2) | 25034 (1.6) |
| COPD | 516 (0.1) | 548 (0.1) | 653 (0.1) | 1925 (0.1) |
| Neoplasms | 3528 (0.5) | 4391 (0.6) | 4827 (0.7) | 13953 (0.9) |

Abbreviations: BP, blood pressure; ESH, European society of hypertension; CVD, cardiovascular disease. IHD, ischemic heart disease; COPD, chronic obstructive pulmonary disease.

**Table S6. Death cases (%) for all-cause and cause-specific mortality in baseline BP group according to 2023 ESH guideline by sex group**

|  | **Optimal (N = 652,400)** | **Normal (N = 700,948)** | **High-normal (N = 674,509)** | **Hypertension (N = 1,571,083)** |
| --- | --- | --- | --- | --- |
| Systolic/diastolic BP, mm Hg | <120/<80 | 120-129/80-84 | 130-139/85-89 | >=140/>=90 |
| **Men** |  |  |  |  |
| Total mortality | 5289 (2.5) | 7029 (2.5) | 8224 (2.9) | 27810 (4.2) |
| CVD mortality | 1528 (0.7) | 2334 (0.8) | 2970 (1.0) | 13025 (2.0) |
| Hemorrhagic Stroke | 239 (0.1) | 399 (0.1) | 569 (0.2) | 2891 (0.4) |
| Ischemic Stroke | 157 (0.1) | 301 (0.1) | 394 (0.1) | 1816 (0.3) |
| IHD | 682 (0.3) | 971 (0.3) | 1207 (0.4) | 4580 (0.7) |
| Non CVD mortality | 3761 (1.8) | 4695 (1.6) | 5254 (1.8) | 14785 (2.3) |
| COPD | 350 (0.2) | 396 (0.1) | 442 (0.2) | 1242 (0.2) |
| Neoplasms | 2139 (1.0) | 2699 (0.9) | 3050 (1.1) | 8251 (1.3) |
| **Woman** |  |  |  |  |
| Total mortality | 3322 (0.8) | 4236 (1.0) | 4868 (1.3) | 19716 (2.2) |
| CVD mortality | 964 (0.2) | 1391 (0.3) | 1771 (0.5) | 9467 (1.0) |
| Hemorrhagic Stroke | 142 (0.0) | 267 (0.1) | 315 (0.1) | 2263 (0.2) |
| Ischemic Stroke | 133 (0.0) | 162 (0.0) | 239 (0.1) | 1258 (0.1) |
| IHD | 388 (0.1) | 550 (0.1) | 724 (0.2) | 3246 (0.4) |
| Non CVD mortality | 2358 (0.5) | 2845 (0.7) | 3097 (0.8) | 10249 (1.1) |
| COPD | 166 (0.0) | 152 (0.0) | 211 (0.1) | 683 (0.1) |
| Neoplasms | 1389 (0.3) | 1692 (0.4) | 1777 (0.5) | 5702 (0.6) |

Abbreviations: BP, blood pressure; ESH, European society of hypertension; CVD, cardiovascular disease. IHD, ischemic heart disease; COPD, chronic obstructive pulmonary disease.

**Table S7. Death cases (%) for all-cause and cause-specific mortality in baseline BP group according to 2023 ESH guideline by age group**

|  | **Optimal (N = 652,400)** | **Normal (N = 700,948)** | **High-normal (N = 674,509)** | **Hypertension (N = 1,571,083)** |
| --- | --- | --- | --- | --- |
| Systolic/diastolic BP, mm Hg | <120/<80 | 120-129/80-84 | 130-139/85-89 | >=140/>=90 |
| **35-44** |  |  |  |  |
| Total mortality | 559 (0.3) | 540 (0.4) | 401 (0.4) | 724 (0.7) |
| CVD mortality | 134 (0.1) | 164 (0.1) | 122 (0.1) | 320 (0.3) |
| Hemorrhagic Stroke | 33 (0.0) | 36 (0.0) | 27 (0.0) | 117 (0.1) |
| Ischemic Stroke | 6 (0.0) | 5 (0.0) | 9 (0.0) | 26 (0.0) |
| IHD | 50 (0.0) | 75 (0.1) | 51 (0.1) | 100 (0.1) |
| Non CVD mortality | 425 (0.2) | 376 (0.3) | 279 (0.3) | 404 (0.4) |
| COPD | 7 (0.0) | 2 (0.0) | 3 (0.0) | 9 (0.0) |
| Neoplasms | 193 (0.1) | 177 (0.1) | 122 (0.1) | 145 (0.1) |
| **45-54** |  |  |  |  |
| Total mortality | 1731 (0.7) | 1817 (0.7) | 1820 (0.8) | 4664 (1.2) |
| CVD mortality | 419 (0.2) | 489 (0.2) | 556 (0.3) | 2157 (0.5) |
| Hemorrhagic Stroke | 69 (0.0) | 109 (0.0) | 131 (0.1) | 696 (0.2) |
| Ischemic Stroke | 41 (0.0) | 49 (0.0) | 48 (0.0) | 190 (0.0) |
| IHD | 184 (0.1) | 193 (0.1) | 240 (0.1) | 723 (0.2) |
| Non CVD mortality | 1312 (0.5) | 1328 (0.5) | 1264 (0.6) | 2507 (0.6) |
| COPD | 49 (0.0) | 31 (0.0) | 31 (0.0) | 74 (0.0) |
| Neoplasms | 755 (0.3) | 719 (0.3) | 652 (0.3) | 1269 (0.3) |
| **55-64** |  |  |  |  |
| Total mortality | 2775 (1.9) | 3497 (1.8) | 4026 (1.9) | 13785 (2.5) |
| CVD mortality | 741 (0.5) | 1084 (0.6) | 1324 (0.6) | 6210 (1.1) |
| Hemorrhagic Stroke | 110 (0.1) | 204 (0.1) | 264 (0.1) | 1545 (0.3) |
| Ischemic Stroke | 85 (0.1) | 125 (0.1) | 174 (0.1) | 812 (0.1) |
| IHD | 329 (0.2) | 425 (0.2) | 556 (0.3) | 2166 (0.4) |
| Non CVD mortality | 2034 (1.4) | 2413 (1.3) | 2702 (1.3) | 7575 (1.4) |
| COPD | 175 (0.1) | 133 (0.1) | 158 (0.1) | 354 (0.1) |
| Neoplasms | 1223 (0.8) | 1519 (0.8) | 1696 (0.8) | 4442 (0.8) |
| **65-75** |  |  |  |  |
| Total mortality | 3546 (4.7) | 5411 (4.5) | 6845 (4.5) | 28353 (5.5) |
| CVD mortality | 1198 (1.6) | 1988 (1.7) | 2739 (1.8) | 13805 (2.7) |
| Hemorrhagic Stroke | 169 (0.2) | 317 (0.3) | 462 (0.3) | 2796 (0.5) |
| Ischemic Stroke | 158 (0.2) | 284 (0.2) | 402 (0.3) | 2046 (0.4) |
| IHD | 507 (0.7) | 828 (0.7) | 1084 (0.7) | 4837 (0.9) |
| Non CVD mortality | 2348 (3.1) | 3423 (2.9) | 4106 (2.7) | 14548 (2.8) |
| COPD | 285 (0.4) | 382 (0.3) | 461 (0.3) | 1488 (0.3) |
| Neoplasms | 1357 (1.8) | 1976 (1.7) | 2357 (1.6) | 8097 (1.6) |

Abbreviations: BP, blood pressure; ESH, European society of hypertension; CVD, cardiovascular disease. IHD, ischemic heart disease; COPD, chronic obstructive pulmonary disease.

**Table S8. Multivariable adjusted hazard ratios for all-cause and cause-specific mortality in baseline BP group according to 2023 ESH guideline by sex group**

|  | **Optimal (N = 652,400)** | **Normal (N = 700,948)** | **High-normal (N = 674,509)** | **Hypertension (N = 1,571,083)** | **P for interaction** |
| --- | --- | --- | --- | --- | --- |
| SBP/DBP, mm Hg | <120 and <80 | 120-129 and 80-84 | 130-139 and/or 85-89 | >=140 and/or >=90 |  |
| **Men** |  |  |  |  |  |
| Total mortality | 1 [Reference] | 0.98 (0.95, 1.02) | 1.02 (0.99, 1.06) | 1.09 (1.05, 1.12) | <0.001 |
| CVD mortality | 1 [Reference] | 1.12 (1.05, 1.19) | 1.26 (1.18, 1.34) | 1.45 (1.37, 1.54) | 0.040 |
| Hemorrhagic Stroke | 1 [Reference] | 1.29 (1.10, 1.51) | 1.69 (1.45, 1.97) | 2.19 (1.90, 2.53) | 0.004 |
| Ischemic Stroke | 1 [Reference] | 1.36 (1.12, 1.65) | 1.53 (1.27, 1.84) | 1.70 (1.43, 2.04) | 0.091 |
| IHD | 1 [Reference] | 1.01 (0.92, 1.12) | 1.10 (1.00, 1.21) | 1.20 (1.10, 1.32) | 0.028 |
| Non CVD mortality | 1 [Reference] | 0.93 (0.89, 0.97) | 0.92 (0.89, 0.96) | 0.94 (0.90, 0.98) | <0.001 |
| COPD | 1 [Reference] | 0.89 (0.77, 1.03) | 0.88 (0.76, 1.01) | 0.94 (0.82, 1.07) | 0.330 |
| Neoplasms | 1 [Reference] | 0.91 (0.86, 0.97) | 0.90 (0.85, 0.95) | 0.88 (0.83, 0.93) | 0.026 |
| **Woman** |  |  |  |  |  |
| Total mortality | 1 [Reference] | 1.05 (1.01, 1.10) | 1.06 (1.01, 1.11) | 1.09 (1.04, 1.13) | <0.001 |
| CVD mortality | 1 [Reference] | 1.15 (1.06, 1.25) | 1.25 (1.15, 1.35) | 1.37 (1.27, 1.47) | 0.040 |
| Hemorrhagic Stroke | 1 [Reference] | 1.65 (1.35, 2.03) | 1.78 (1.45, 2.17) | 2.48 (2.06, 2.99) | 0.004 |
| Ischemic Stroke | 1 [Reference] | 0.91 (0.72, 1.14) | 1.09 (0.88, 1.35) | 1.17 (0.96, 1.43) | 0.091 |
| IHD | 1 [Reference] | 1.08 (0.95, 1.23) | 1.18 (1.04, 1.34) | 1.16 (1.03, 1.31) | 0.028 |
| Non CVD mortality | 1 [Reference] | 1.02 (0.97, 1.08) | 0.99 (0.93, 1.04) | 0.98 (0.93, 1.03) | <0.001 |
| COPD | 1 [Reference] | 0.72 (0.58, 0.89) | 0.85 (0.69, 1.05) | 0.79 (0.65, 0.96) | 0.330 |
| Neoplasms | 1 [Reference] | 1.02 (0.95, 1.10) | 0.95 (0.89, 1.03) | 0.96 (0.89, 1.02) | 0.026 |

Abbreviations: BP, blood pressure; ESH, European society of hypertension; CVD, cardiovascular disease. IHD, ischemic heart disease; COPD, chronic obstructive pulmonary disease.

**Table S9. Multivariable adjusted hazard ratios for all-cause and cause-specific mortality in baseline BP group according to 2023 ESH guideline by age group**

|  | **Optimal (N = 652,400)** | **Normal (N = 700,948)** | **High-normal (N = 674,509)** | **Hypertension (N = 1,571,083)** | **P for interaction** |
| --- | --- | --- | --- | --- | --- |
| SBP/DBP, mm Hg | <120 and <80 | 120-129 and 80-84 | 130-139 and/or 85-89 | >=140 and/or >=90 |  |
| **35-44** |  |  |  |  |  |
| Total mortality | 1 [Reference] | 1.10 (0.98, 1.25) | 1.17 (1.02, 1.33) | 1.46 (1.28, 1.67) | <0.001 |
| CVD mortality | 1 [Reference] | 1.32 (1.05, 1.67) | 1.37 (1.06, 1.76) | 1.91 (1.49, 2.46) | <0.001 |
| Hemorrhagic Stroke | 1 [Reference] | 1.23 (0.77, 1.97) | 1.31 (0.78, 2.20) | 2.81 (1.77, 4.45) | <0.001 |
| Ischemic Stroke | 1 [Reference] | 0.94 (0.28, 3.10) | 2.65 (0.94, 7.49) | 4.02 (1.51, 10.68) | <0.001 |
| IHD | 1 [Reference] | 1.46 (1.02, 2.11) | 1.31 (0.87, 1.96) | 1.55 (1.03, 2.34) | <0.001 |
| Non CVD mortality | 1 [Reference] | 1.04 (0.90, 1.20) | 1.11 (0.95, 1.30) | 1.32 (1.12, 1.55) | <0.001 |
| COPD | 1 [Reference] | 0.34 (0.06, 1.83) | 0.72 (0.19, 2.69) | 1.68 (0.50, 5.58) | 0.001 |
| Neoplasms | 1 [Reference] | 1.12 (0.91, 1.38) | 1.14 (0.91, 1.45) | 1.18 (0.91, 1.52) | 0.038 |
| **45-54** |  |  |  |  |  |
| Total mortality | 1 [Reference] | 1.00 (0.93, 1.06) | 1.10 (1.03, 1.17) | 1.19 (1.11, 1.26) | <0.001 |
| CVD mortality | 1 [Reference] | 1.10 (0.96, 1.25) | 1.37 (1.21, 1.56) | 1.73 (1.54, 1.95) | <0.001 |
| Hemorrhagic Stroke | 1 [Reference] | 1.54 (1.14, 2.09) | 2.09 (1.56, 2.79) | 2.89 (2.20, 3.78) | <0.001 |
| Ischemic Stroke | 1 [Reference] | 1.09 (0.72, 1.66) | 1.17 (0.77, 1.78) | 1.39 (0.94, 2.06) | <0.001 |
| IHD | 1 [Reference] | 0.94 (0.77, 1.15) | 1.26 (1.03, 1.53) | 1.49 (1.24, 1.79) | <0.001 |
| Non CVD mortality | 1 [Reference] | 0.96 (0.89, 1.04) | 1.01 (0.93, 1.09) | 1.01 (0.93, 1.09) | <0.001 |
| COPD | 1 [Reference] | 0.63 (0.40, 0.98) | 0.72 (0.46, 1.14) | 0.70 (0.46, 1.08) | 0.001 |
| Neoplasms | 1 [Reference] | 0.91 (0.82, 1.01) | 0.91 (0.82, 1.01) | 0.91 (0.82, 1.02) | 0.038 |
| **55-64** |  |  |  |  |  |
| Total mortality | 1 [Reference] | 0.97 (0.93, 1.02) | 1.00 (0.95, 1.05) | 1.06 (1.01, 1.11) | <0.001 |
| CVD mortality | 1 [Reference] | 1.12 (1.02, 1.23) | 1.23 (1.12, 1.34) | 1.41 (1.30, 1.54) | <0.001 |
| Hemorrhagic Stroke | 1 [Reference] | 1.46 (1.16, 1.85) | 1.72 (1.37, 2.15) | 2.27 (1.85, 2.79) | <0.001 |
| Ischemic Stroke | 1 [Reference] | 1.11 (0.84, 1.46) | 1.36 (1.05, 1.77) | 1.41 (1.10, 1.81) | <0.001 |
| IHD | 1 [Reference] | 0.97 (0.84, 1.12) | 1.12 (0.98, 1.29) | 1.18 (1.03, 1.34) | <0.001 |
| Non CVD mortality | 1 [Reference] | 0.92 (0.87, 0.98) | 0.92 (0.87, 0.98) | 0.94 (0.89, 0.99) | <0.001 |
| COPD | 1 [Reference] | 0.65 (0.52, 0.82) | 0.74 (0.59, 0.92) | 0.68 (0.55, 0.84) | 0.001 |
| Neoplasms | 1 [Reference] | 0.95 (0.88, 1.03) | 0.95 (0.88, 1.02) | 0.94 (0.88, 1.01) | 0.038 |
| **65-75** |  |  |  |  |  |
| Total mortality | 1 [Reference] | 1.00 (0.96, 1.04) | 0.98 (0.95, 1.03) | 1.01 (0.97, 1.05) | <0.001 |
| CVD mortality | 1 [Reference] | 1.08 (1.01, 1.16) | 1.15 (1.08, 1.24) | 1.24 (1.16, 1.32) | <0.001 |
| Hemorrhagic Stroke | 1 [Reference] | 1.26 (1.05, 1.52) | 1.45 (1.21, 1.73) | 1.79 (1.52, 2.12) | <0.001 |
| Ischemic Stroke | 1 [Reference] | 1.16 (0.95, 1.41) | 1.26 (1.05, 1.51) | 1.38 (1.16, 1.65) | <0.001 |
| IHD | 1 [Reference] | 1.04 (0.93, 1.16) | 1.04 (0.94, 1.16) | 1.06 (0.96, 1.17) | <0.001 |
| Non CVD mortality | 1 [Reference] | 0.96 (0.91, 1.01) | 0.90 (0.86, 0.95) | 0.90 (0.85, 0.94) | <0.001 |
| COPD | 1 [Reference] | 0.98 (0.84, 1.14) | 0.96 (0.83, 1.12) | 1.01 (0.88, 1.16) | 0.001 |
| Neoplasms | 1 [Reference] | 0.94 (0.88, 1.01) | 0.87 (0.81, 0.93) | 0.85 (0.80, 0.91) | 0.038 |

Abbreviations: BP, blood pressure; ESH, European society of hypertension; CVD, cardiovascular disease. IHD, ischemic heart disease; COPD, chronic obstructive pulmonary disease. NA: model does not converge.

**Table S10. Comparison of results between the main model and the model after excluding the first 3 years of death (New model) in baseline BP group**

| **Outcome** | **BP group** | **HR (95%CI)** | | **Absolute change for HR** |
| --- | --- | --- | --- | --- |
|  |  | **Main model** | **New model** |  |
| Total mortality | Normal | 1.02 (0.99, 1.04) | 1.01 (0.97, 1.05) | -0.01 |
| Total mortality | High-normal | 1.04 (1.01, 1.07) | 1.04 (1.00, 1.08) | -0.00 |
| Total mortality | Hypertension | 1.09 (1.07, 1.12) | 1.16 (1.12, 1.21) | -0.04 |
| CVD mortality | Normal | 1.14 (1.09, 1.20) | 1.12 (1.05, 1.20) | -0.02 |
| CVD mortality | High-normal | 1.28 (1.21, 1.34) | 1.28 (1.21, 1.36) | 0.01 |
| CVD mortality | Hypertension | 1.44 (1.38, 1.51) | 1.64 (1.54, 1.75) | -0.07 |
| Hemorrhagic Stroke | Normal | 1.43 (1.26, 1.63) | 1.38 (1.18, 1.63) | -0.05 |
| Hemorrhagic Stroke | High-normal | 1.75 (1.55, 1.98) | 1.76 (1.50, 2.05) | 0.01 |
| Hemorrhagic Stroke | Hypertension | 2.33 (2.08, 2.61) | 2.88 (2.47, 3.37) | -0.18 |
| Ischemic Stroke | Normal | 1.17 (1.01, 1.35) | 1.18 (0.97, 1.44) | 0.02 |
| Ischemic Stroke | High-normal | 1.35 (1.17, 1.55) | 1.53 (1.27, 1.84) | 0.19 |
| Ischemic Stroke | Hypertension | 1.49 (1.30, 1.70) | 1.77 (1.46, 2.14) | -0.01 |
| IHD | Normal | 1.05 (0.97, 1.14) | 1.05 (0.95, 1.15) | -0.01 |
| IHD | High-normal | 1.15 (1.07, 1.24) | 1.14 (1.04, 1.25) | -0.01 |
| IHD | Hypertension | 1.21 (1.13, 1.30) | 1.31 (1.18, 1.44) | -0.05 |
| Non CVD mortality | Normal | 0.97 (0.93, 1.00) | 0.97 (0.93, 1.01) | -0.00 |
| Non CVD mortality | High-normal | 0.95 (0.92, 0.98) | 0.94 (0.91, 0.99) | -0.01 |
| Non CVD mortality | Hypertension | 0.96 (0.93, 0.99) | 0.94 (0.90, 0.99) | -0.03 |
| COPD | Normal | 0.84 (0.74, 0.94) | 0.86 (0.73, 1.01) | 0.02 |
| COPD | High-normal | 0.87 (0.77, 0.98) | 0.88 (0.75, 1.03) | 0.00 |
| COPD | Hypertension | 0.89 (0.80, 0.99) | 0.95 (0.80, 1.15) | 0.05 |
| Neoplasms | Normal | 0.95 (0.91, 0.99) | 0.94 (0.89, 0.99) | -0.01 |
| Neoplasms | High-normal | 0.91 (0.88, 0.96) | 0.91 (0.86, 0.96) | -0.01 |
| Neoplasms | Hypertension | 0.90 (0.87, 0.94) | 0.84 (0.79, 0.90) | -0.05 |

Abbreviations: BP, blood pressure; CVD, cardiovascular disease. IHD, ischemic heart disease; COPD, chronic obstructive pulmonary disease; HR: hazard ratio.

**Table S11. Comparison of results between the main model and the model additionally adjustment for diet and physical activity (New model) in baseline BP group**

| **Outcome** | **BP group** | **HR (95%CI)** | | **Absolute change for HR** |
| --- | --- | --- | --- | --- |
|  |  | **Main model** | **New model** |  |
| Total mortality | Normal | 1.02 (0.99, 1.04) | 1.02 (0.97, 1.06) | 0.00 |
| Total mortality | High-normal | 1.04 (1.01, 1.07) | 1.02 (0.97, 1.07) | 0.02 |
| Total mortality | Hypertension | 1.09 (1.07, 1.12) | 1.04 (1.00, 1.09) | 0.05 |
| CVD mortality | Normal | 1.14 (1.09, 1.20) | 1.16 (1.06, 1.26) | -0.01 |
| CVD mortality | High-normal | 1.28 (1.21, 1.34) | 1.22 (1.12, 1.33) | 0.06 |
| CVD mortality | Hypertension | 1.44 (1.38, 1.51) | 1.28 (1.18, 1.39) | 0.16 |
| Hemorrhagic Stroke | Normal | 1.43 (1.26, 1.63) | 1.53 (1.25, 1.89) | -0.10 |
| Hemorrhagic Stroke | High-normal | 1.75 (1.55, 1.98) | 1.72 (1.40, 2.10) | 0.03 |
| Hemorrhagic Stroke | Hypertension | 2.33 (2.08, 2.61) | 2.08 (1.70, 2.53) | 0.25 |
| Ischemic Stroke | Normal | 1.17 (1.01, 1.35) | 1.23 (0.97, 1.56) | -0.06 |
| Ischemic Stroke | High-normal | 1.35 (1.17, 1.55) | 1.20 (0.95, 1.51) | 0.15 |
| Ischemic Stroke | Hypertension | 1.49 (1.30, 1.70) | 1.24 (0.99, 1.57) | 0.24 |
| IHD | Normal | 1.05 (0.97, 1.14) | 0.99 (0.87, 1.13) | 0.06 |
| IHD | High-normal | 1.15 (1.07, 1.24) | 1.11 (0.98, 1.26) | 0.04 |
| IHD | Hypertension | 1.21 (1.13, 1.30) | 1.07 (0.94, 1.22) | 0.14 |
| Non CVD mortality | Normal | 0.97 (0.93, 1.00) | 0.96 (0.91, 1.02) | 0.00 |
| Non CVD mortality | High-normal | 0.95 (0.92, 0.98) | 0.94 (0.89, 1.00) | 0.01 |
| Non CVD mortality | Hypertension | 0.96 (0.93, 0.99) | 0.95 (0.90, 1.00) | 0.01 |
| COPD | Normal | 0.84 (0.74, 0.94) | 0.88 (0.72, 1.09) | -0.05 |
| COPD | High-normal | 0.87 (0.77, 0.98) | 1.04 (0.86, 1.27) | -0.17 |
| COPD | Hypertension | 0.89 (0.80, 0.99) | 1.02 (0.84, 1.24) | -0.13 |
| Neoplasms | Normal | 0.95 (0.91, 0.99) | 0.96 (0.90, 1.04) | -0.01 |
| Neoplasms | High-normal | 0.91 (0.88, 0.96) | 0.88 (0.82, 0.95) | 0.03 |
| Neoplasms | Hypertension | 0.90 (0.87, 0.94) | 0.90 (0.84, 0.97) | 0.00 |

Abbreviations: BP, blood pressure; CVD, cardiovascular disease. IHD, ischemic heart disease; COPD, chronic obstructive pulmonary disease, HR: hazard ratio.

**Table S12. Death cases (%) for all-cause and cause-specific disease in** **BP trajectory pattern during the follow-up**

|  | **Optimal-stable (N = 20,419)** | **Normal-stable (N =30,606)** | **High-normal-stable (N = 23,363)** | **High-normal-increasing (N =3,742)** |
| --- | --- | --- | --- | --- |
| Total mortality | 256 (1.3) | 503 (1.6) | 452 (1.9) | 122 (3.3) |
| CVD mortality | 96 (0.5) | 190 (0.6) | 196 (0.8) | 60 (1.6) |
| Hemorrhagic Stroke | 10 (0.0) | 30 (0.1) | 37 (0.2) | 14 (0.4) |
| Ischemic Stroke | 7 (0.0) | 14 (0.0) | 34 (0.1) | 9 (0.2) |
| IHD | 45 (0.2) | 100 (0.3) | 77 (0.3) | 26 (0.7) |
| Non CVD mortality | 160 (0.8) | 313 (1.0) | 256 (1.1) | 62 (1.7) |
| COPD | 6 (0.0) | 28 (0.1) | 14 (0.1) | 3 (0.1) |
| Neoplasms | 104 (0.5) | 176 (0.6) | 157 (0.7) | 35 (0.9) |

Abbreviations: BP, blood pressure; ESH, European society of hypertension; CVD, cardiovascular disease. IHD, ischemic heart disease; COPD, chronic obstructive pulmonary disease.

**Table S13. Death cases (%) for all-cause and cause-specific mortality in BP trajectory pattern during the follow-up by sex group**

|  | **Optimal-stable (N = 20,419)** | **Normal-stable (N =30,606)** | **High-normal-stable (N = 23,363)** | **High-normal-increasing (N =3,742)** |
| --- | --- | --- | --- | --- |
| **Men** |  |  |  |  |
| Total mortality | 163 (2.5) | 320 (2.6) | 290 (2.9) | 74 (5.2) |
| CVD mortality | 65 (1.0) | 134 (1.1) | 138 (1.4) | 34 (2.4) |
| Hemorrhagic Stroke | 6 (0.1) | 23 (0.2) | 30 (0.3) | 9 (0.6) |
| Ischemic Stroke | 5 (0.1) | 5 (0.0) | 23 (0.2) | 5 (0.3) |
| IHD | 28 (0.4) | 73 (0.6) | 54 (0.5) | 17 (1.2) |
| Non CVD mortality | 98 (1.5) | 186 (1.5) | 152 (1.5) | 40 (2.8) |
| COPD | 4 (0.1) | 17 (0.1) | 10 (0.1) | 3 (0.2) |
| Neoplasms | 65 (1.0) | 103 (0.8) | 93 (0.9) | 22 (1.5) |
| **Woman** |  |  |  |  |
| Total mortality | 93 (0.7) | 183 (1.0) | 162 (1.2) | 48 (2.1) |
| CVD mortality | 31 (0.2) | 56 (0.3) | 58 (0.4) | 26 (1.1) |
| Hemorrhagic Stroke | 4 (0.0) | 7 (0.0) | 7 (0.1) | 5 (0.2) |
| Ischemic Stroke | 2 (0.0) | 9 (0.0) | 11 (0.1) | 4 (0.2) |
| IHD | 17 (0.1) | 27 (0.1) | 23 (0.2) | 9 (0.4) |
| Non CVD mortality | 62 (0.4) | 127 (0.7) | 104 (0.8) | 22 (1.0) |
| COPD | 2 (0.0) | 11 (0.1) | 4 (0.0) | 0 (0.0) |
| Neoplasms | 39 (0.3) | 73 (0.4) | 64 (0.5) | 13 (0.6) |

Abbreviations: BP, blood pressure; CVD, cardiovascular disease. IHD, ischemic heart disease; COPD, chronic obstructive pulmonary disease.

**Table S14. Death cases (%) for all-cause and cause-specific mortality in BP trajectory pattern during the follow-up by age group**

|  | **Optimal-stable (N = 20,419)** | **Normal-stable (N =30,606)** | **High-normal-stable (N = 23,363)** | **High-normal-increasing (N =3,742)** |
| --- | --- | --- | --- | --- |
| **35-44** |  |  |  |  |
| Total mortality | 19 (0.3) | 23 (0.5) | 11 (0.6) | 2 (1.3) |
| CVD mortality | 7 (0.1) | 7 (0.1) | 2 (0.1) | 1 (0.6) |
| Hemorrhagic Stroke | 2 (0.0) | 2 (0.0) | 1 (0.1) | 0 (0.0) |
| Ischemic Stroke | 0 (0.0) | 0 (0.0) | 0 (0.0) | 0 (0.0) |
| IHD | 4 (0.1) | 2 (0.0) | 0 (0.0) | 0 (0.0) |
| Non CVD mortality | 12 (0.2) | 16 (0.3) | 9 (0.5) | 1 (0.6) |
| COPD | 0 (0.0) | 0 (0.0) | 0 (0.0) | 0 (0.0) |
| Neoplasms | 6 (0.1) | 7 (0.1) | 6 (0.3) | 0 (0.0) |
| **45-54** |  |  |  |  |
| Total mortality | 44 (0.5) | 81 (0.8) | 50 (0.8) | 9 (1.3) |
| CVD mortality | 14 (0.2) | 30 (0.3) | 16 (0.3) | 4 (0.6) |
| Hemorrhagic Stroke | 3 (0.0) | 5 (0.0) | 4 (0.1) | 1 (0.1) |
| Ischemic Stroke | 1 (0.0) | 1 (0.0) | 2 (0.0) | 1 (0.1) |
| IHD | 5 (0.1) | 18 (0.2) | 7 (0.1) | 2 (0.3) |
| Non CVD mortality | 30 (0.4) | 51 (0.5) | 34 (0.5) | 5 (0.7) |
| COPD | 0 (0.0) | 3 (0.0) | 1 (0.0) | 0 (0.0) |
| Neoplasms | 20 (0.2) | 28 (0.3) | 17 (0.3) | 4 (0.6) |
| **55-64** |  |  |  |  |
| Total mortality | 95 (1.9) | 184 (1.8) | 151 (1.7) | 38 (2.6) |
| CVD mortality | 30 (0.6) | 65 (0.6) | 60 (0.7) | 15 (1.0) |
| Hemorrhagic Stroke | 2 (0.0) | 10 (0.1) | 15 (0.2) | 3 (0.2) |
| Ischemic Stroke | 2 (0.0) | 7 (0.1) | 11 (0.1) | 3 (0.2) |
| IHD | 14 (0.3) | 32 (0.3) | 23 (0.3) | 4 (0.3) |
| Non CVD mortality | 65 (1.3) | 119 (1.2) | 91 (1.0) | 23 (1.6) |
| COPD | 3 (0.1) | 10 (0.1) | 4 (0.0) | 2 (0.1) |
| Neoplasms | 44 (0.9) | 69 (0.7) | 59 (0.7) | 11 (0.8) |
| **65-75** |  |  |  |  |
| Total mortality | 98 (5.6) | 215 (4.3) | 240 (3.9) | 73 (5.0) |
| CVD mortality | 45 (2.6) | 88 (1.8) | 118 (1.9) | 40 (2.7) |
| Hemorrhagic Stroke | 3 (0.2) | 13 (0.3) | 17 (0.3) | 10 (0.7) |
| Ischemic Stroke | 4 (0.2) | 6 (0.1) | 21 (0.3) | 5 (0.3) |
| IHD | 22 (1.3) | 48 (1.0) | 47 (0.8) | 20 (1.4) |
| Non CVD mortality | 53 (3.1) | 127 (2.5) | 122 (2.0) | 33 (2.3) |
| COPD | 3 (0.2) | 15 (0.3) | 9 (0.1) | 1 (0.1) |
| Neoplasms | 34 (2.0) | 72 (1.4) | 75 (1.2) | 20 (1.4) |

Abbreviations: BP, blood pressure; CVD, cardiovascular disease. IHD, ischemic heart disease; COPD, chronic obstructive pulmonary disease.

**Table S15. Multivariable adjusted hazard ratios for all-cause and cause-specific mortality in BP trajectory pattern during the follow-up by sex group**

|  | **Optimal-stable (N = 20,419)** | **Normal-stable (N =30,606)** | **High-normal-stable (N = 23,363)** | **High-normal-increasing (N =3,742)** | **P for interaction** |
| --- | --- | --- | --- | --- | --- |
| **Men** |  |  |  |  |  |
| Total mortality | 1 [Reference] | 0.91 (0.75, 1.12) | 0.88 (0.72, 1.09) | 1.32 (0.99, 1.78) | 0.910 |
| CVD mortality | 1 [Reference] | 0.99 (0.73, 1.37) | 1.07 (0.78, 1.48) | 1.41 (0.90, 2.23) | 0.386 |
| Hemorrhagic Stroke | 1 [Reference] | 1.79 (0.67, 4.84) | 2.59 (0.98, 6.89) | 4.23 (1.31, 13.74) | 0.556 |
| Ischemic Stroke | 1 [Reference] | 0.54 (0.12, 2.45) | 2.81 (0.82, 9.66) | 3.40 (0.79, 14.80) | 0.273 |
| IHD | 1 [Reference] | 1.37 (0.86, 2.22) | 0.99 (0.60, 1.67) | 1.71 (0.88, 3.35) | 0.684 |
| Non CVD mortality | 1 [Reference] | 0.86 (0.67, 1.12) | 0.76 (0.58, 1.01) | 1.27 (0.86, 1.87) | 0.347 |
| COPD | 1 [Reference] | 2.43 (0.70, 8.54) | 1.40 (0.37, 5.37) | 3.02 (0.59, 15.66) | 0.336 |
| Neoplasms | 1 [Reference] | 0.71 (0.51, 1.00) | 0.68 (0.49, 0.97) | 1.00 (0.60, 1.68) | 0.542 |
| **Woman** |  |  |  |  |  |
| Total mortality | 1 [Reference] | 0.92 (0.70, 1.21) | 0.93 (0.71, 1.24) | 1.34 (0.92, 1.97) | 0.910 |
| CVD mortality | 1 [Reference] | 0.78 (0.49, 1.24) | 0.80 (0.51, 1.29) | 1.61 (0.92, 2.85) | 0.386 |
| Hemorrhagic Stroke | 1 [Reference] | 0.96 (0.28, 3.41) | 0.92 (0.24, 3.54) | 2.86 (0.64, 12.88) | 0.556 |
| Ischemic Stroke | 1 [Reference] | 1.53 (0.32, 7.34) | 2.01 (0.43, 9.45) | 2.78 (0.48, 16.19) | 0.273 |
| IHD | 1 [Reference] | 0.75 (0.40, 1.45) | 0.53 (0.27, 1.07) | 1.05 (0.44, 2.51) | 0.684 |
| Non CVD mortality | 1 [Reference] | 1.00 (0.72, 1.41) | 1.01 (0.72, 1.45) | 1.10 (0.65, 1.86) | 0.347 |
| COPD | 1 [Reference] | 4.28 (1.32, 13.93) | 1.42 (0.39, 5.15) | 0.00 (0.00, Inf) | 0.336 |
| Neoplasms | 1 [Reference] | 0.94 (0.62, 1.45) | 1.03 (0.66, 1.61) | 1.02 (0.52, 2.02) | 0.542 |

Abbreviations: BP, blood pressure; CVD, cardiovascular disease. IHD, ischemic heart disease; COPD, chronic obstructive pulmonary disease. NA: model does not converge.

**Table S16. Multivariable adjusted hazard ratios for all-cause and cause-specific mortality in BP trajectory pattern during the follow-up by age group**

|  | **Optimal-stable (N = 20,419)** | **Normal-stable (N =30,606)** | **High-normal-stable (N = 23,363)** | **High-normal-increasing (N =3,742)** | **P for interaction** |
| --- | --- | --- | --- | --- | --- |
| **35-44** |  |  |  |  |  |
| Total mortality | 1 [Reference] | 0.93 (0.47, 1.88) | 0.98 (0.42, 2.34) | 3.05 (0.68, 13.73) | 0.475 |
| CVD mortality | 1 [Reference] | 0.53 (0.15, 1.92) | 0.20 (0.03, 1.77) | 3.77 (0.41, 34.83) | 0.583 |
| Hemorrhagic Stroke | 1 [Reference] | 0.12 (0.02, 1.29) | NA | NA | 0.746 |
| Ischemic Stroke | 1 [Reference] | NA | NA | NA | 0.757 |
| IHD | 1 [Reference] | 0.11 (0.02, 0.67) | NA | NA | 0.639 |
| Non CVD mortality | 1 [Reference] | 1.22 (0.52, 2.89) | 1.66 (0.61, 4.53) | 2.79 (0.35, 22.61) | 0.387 |
| COPD | 1 [Reference] | NA | NA | NA | 0.882 |
| Neoplasms | 1 [Reference] | 1.38 (0.38, 5.06) | 3.22 (0.82, 12.75) | NA | 0.516 |
| **45-54** |  |  |  |  |  |
| Total mortality | 1 [Reference] | 1.16 (0.79, 1.74) | 1.29 (0.84, 2.01) | 2.07 (0.99, 4.37) | 0.475 |
| CVD mortality | 1 [Reference] | 1.26 (0.66, 2.44) | 1.20 (0.57, 2.52) | 2.43 (0.76, 7.76) | 0.583 |
| Hemorrhagic Stroke | 1 [Reference] | 0.99 (0.22, 4.52) | 1.64 (0.35, 7.79) | 4.17 (0.39, 45.01) | 0.746 |
| Ischemic Stroke | 1 [Reference] | NA | 1.47 (0.20, 10.92) | NA | 0.757 |
| IHD | 1 [Reference] | 2.29 (0.84, 6.32) | 1.36 (0.42, 4.45) | 2.87 (0.52, 15.88) | 0.639 |
| Non CVD mortality | 1 [Reference] | 1.10 (0.67, 1.83) | 1.35 (0.79, 2.31) | 1.91 (0.72, 5.09) | 0.387 |
| COPD | 1 [Reference] | NA | NA | NA | 0.882 |
| Neoplasms | 1 [Reference] | 0.92 (0.48, 1.79) | 1.04 (0.51, 2.15) | 2.49 (0.81, 7.69) | 0.516 |
| **55-64** |  |  |  |  |  |
| Total mortality | 1 [Reference] | 0.95 (0.73, 1.25) | 0.88 (0.67, 1.17) | 1.53 (1.03, 2.27) | 0.475 |
| CVD mortality | 1 [Reference] | 1.21 (0.76, 1.95) | 1.14 (0.70, 1.88) | 1.89 (0.98, 3.66) | 0.583 |
| Hemorrhagic Stroke | 1 [Reference] | NA | NA | NA | 0.746 |
| Ischemic Stroke | 1 [Reference] | NA | NA | NA | 0.757 |
| IHD | 1 [Reference] | 1.28 (0.64, 2.58) | 0.89 (0.42, 1.91) | 1.08 (0.34, 3.46) | 0.639 |
| Non CVD mortality | 1 [Reference] | 0.84 (0.61, 1.18) | 0.77 (0.55, 1.10) | 1.37 (0.84, 2.25) | 0.387 |
| COPD | 1 [Reference] | NA | 1.68 (0.17, 17.01) | NA | 0.882 |
| Neoplasms | 1 [Reference] | 0.73 (0.49, 1.11) | 0.72 (0.47, 1.10) | 0.94 (0.48, 1.87) | 0.516 |
| **65-75** |  |  |  |  |  |
| Total mortality | 1 [Reference] | 0.77 (0.60, 0.99) | 0.78 (0.61, 1.00) | 1.01 (0.73, 1.40) | 0.475 |
| CVD mortality | 1 [Reference] | 0.71 (0.49, 1.05) | 0.86 (0.60, 1.25) | 1.18 (0.75, 1.88) | 0.583 |
| Hemorrhagic Stroke | 1 [Reference] | 1.35 (0.38, 4.86) | 1.61 (0.46, 5.63) | 3.11 (0.79, 12.35) | 0.746 |
| Ischemic Stroke | 1 [Reference] | 0.59 (0.14, 2.53) | 2.02 (0.59, 6.96) | 2.19 (0.50, 9.63) | 0.757 |
| IHD | 1 [Reference] | 0.89 (0.52, 1.54) | 0.71 (0.41, 1.24) | 1.33 (0.69, 2.60) | 0.639 |
| Non CVD mortality | 1 [Reference] | 0.82 (0.59, 1.14) | 0.71 (0.51, 1.00) | 0.87 (0.56, 1.39) | 0.387 |
| COPD | 1 [Reference] | 1.54 (0.43, 5.61) | 0.99 (0.26, 3.91) | 0.58 (0.06, 5.87) | 0.882 |
| Neoplasms | 1 [Reference] | 0.72 (0.48, 1.12) | 0.68 (0.45, 1.06) | 0.81 (0.45, 1.47) | 0.516 |

Abbreviations: BP, blood pressure; CVD, cardiovascular disease. IHD, ischemic heart disease; COPD, chronic obstructive pulmonary disease. NA: model does not converge.


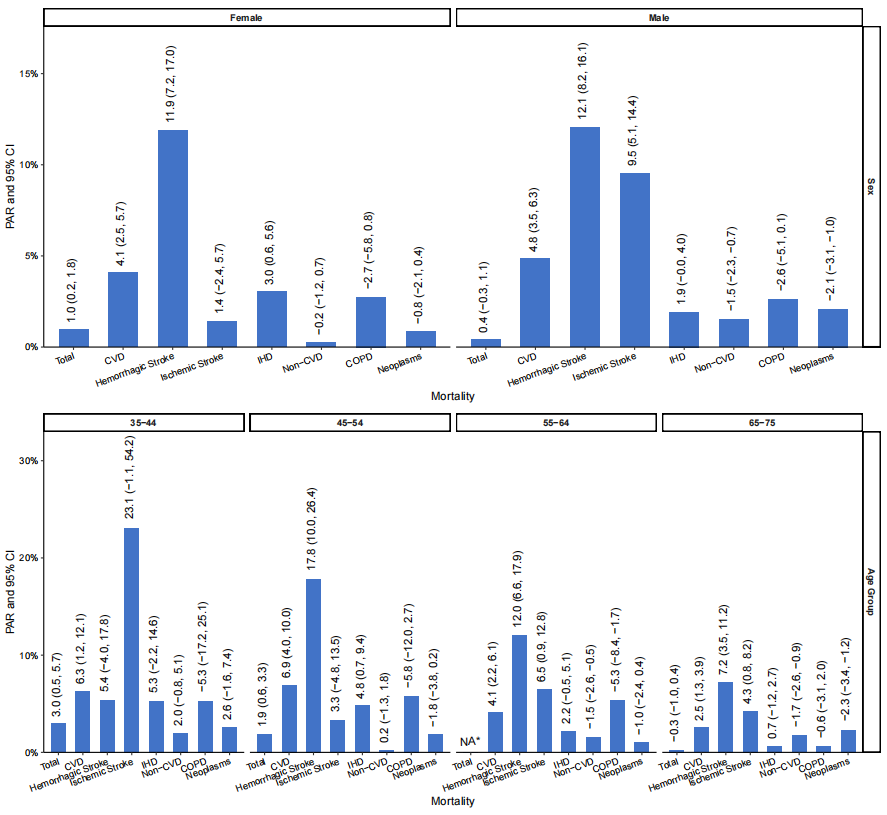


**Figure S2. Population attributable risk due to "High-normal" in baseline BP group by sex and age group**

Abbreviations: PAR, population attributable risk; BP, blood pressure; CVD, cardiovascular disease; IHD, ischemic heart disease; COPD, chronic obstructive pulmonary disease.

*NA represents the PAR value is too small to be estimated.


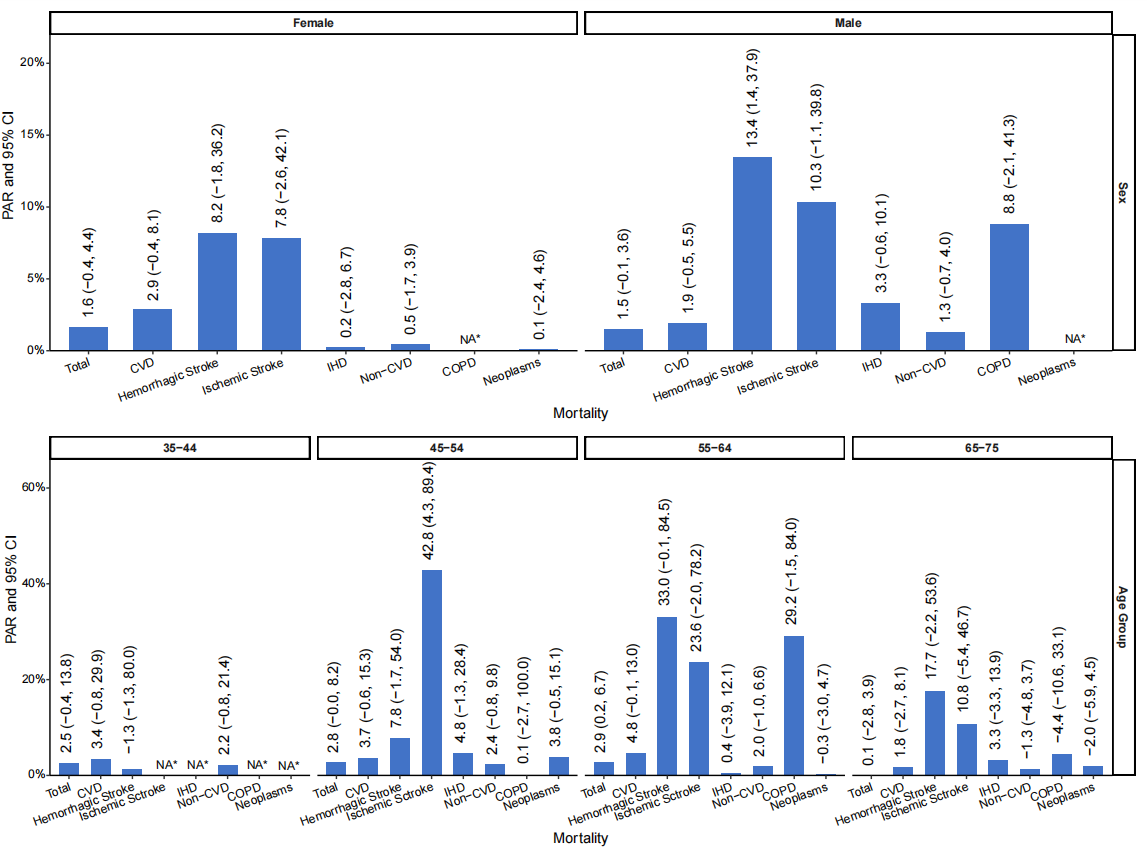


**Figure S3. Population attributable risk due to "High normal-increasing" in BP trajectory pattern during the follow-up by sex and age group**

Abbreviations: PAR, population attributable risk; BP, blood pressure; CVD, cardiovascular disease; IHD, ischemic heart disease; COPD, chronic obstructive pulmonary disease.

*NA represents the PAR value is too small to be estimated.
